# Small RNA sequencing in individually selected sperm: Biomarkers for male subfertility and predictors of pregnancy success

**DOI:** 10.1016/j.ncrna.2025.09.008

**Published:** 2025-10-10

**Authors:** Mohammad A. Al Smadi, Haidar Haidar, Albert Salas-Huetos, Ulrike Fischer, Hashim Abdul-Khaliq, Eckart Meese, Masood Abu-Halima

**Affiliations:** a Reproductive Endocrinology and IVF Unit, King Hussein Medical Centre, Amman, Jordan; b Institute of Human Genetics, Saarland University, Homburg, Germany; c Universitat Rovira i Virgili, Departament de Ciències Mèdiques Bàsiques, Unitat de Medicina Preventiva, Alimentació, Nutrició, Desenvolupament i Salut Mental (ANUT-DSM), Reus, Spain; d Centro de Investigación Biomédica en Red de Fisiopatología de la Obesidad y Nutrición, Instituto de Salud Carlos III, Madrid, Spain; e Department of Paediatric Cardiology, Saarland University Hospital, Homburg, Germany

**Keywords:** microRNA, Small RNAs, Sequencing, Single sperm, Male infertility

## Abstract

**Background:**

Sperm quality defined by motility and morphology has critical implications for fertility and pregnancy outcomes. Small RNAs, including microRNAs (miRNAs) and Piwi-interacting RNAs (piRNAs), play regulatory roles and may reflect underlying sperm function. This study aimed to identify small RNA types in sperm based on motility and morphology, examine their correlation with sperm and clinical parameters, and develop diagnostic tools to predict pregnancy outcomes.

**Methods:**

A total of 98 male partners of couples undergoing infertility treatment were included. Thirteen males provided 39 sperm samples categorized into three groups based on quality: A (good), B (intermediate), and C (poor), each with 1500 individually selected sperm. Additionally, 85 males contributed purified sperm samples with various spermatogenic impairments. Small RNA sequencing was performed followed by RT-qPCR validation.

**Results:**

Small RNA sequencing revealed a diverse RNA landscape in sperm, with long non-coding RNA (lncRNA) being the most abundant. Regulatory RNAs such as miRNAs and piRNAs were present at varying levels. Differential expression analysis identified 16 miRNAs and 37 piRNAs significantly different between groups A and C. Strong correlations were observed between miRNA/piRNA expression and sperm motility and morphology in groups A and C, but not in group B. miRNA expression levels were associated with sperm quality and pregnancy outcomes, including embryo quality, β-hCG levels, and live birth. Notably, hsa-miR-15b-5p, hsa-miR-19a-5p, and hsa-miR-20a-5p were linked to sperm impairments and hormonal markers (β-hCG, FSH, and LH). Higher expression of these miRNAs was associated with negative β-hCG outcomes and poor IVF prognosis. Lower expression of hsa-miR-15b-5p and hsa-miR-20a-5p was found in G1 embryos compared to G2 embryos. These miRNAs were also significantly correlated with live birth outcomes: higher expression was linked to failed IVF, while lower expression was linked to successful live births. Diagnostic validation showed AUCs of 0.76, 0.71, and 0.74 for hsa-miR-15b-5p, hsa-miR-19a-5p, and hsa-miR-20a-5p, respectively. A combined model yielded an AUC of 0.75.

**Conclusion:**

These findings suggest that hsa-miR-15b-5p, hsa-miR-19a-5p, and hsa-miR-20a-5p could serve as potential biomarkers for assessing sperm quality and predicting pregnancy outcomes.

## 1 Introduction

Infertility is a significant public health issue that occurs in about 15 % of couples [1,2]. It is the inability to conceive after at least one year of normal, unprotected intercourse. Male factors account for approximately 40–50 % of infertility cases and are usually coupled with aberrant sperm development, low sperm motility, or defective function [1]. These defects may be due to congenital defects of the reproductive tract, genetic abnormalities, endocrine disorders, or exposure to toxic environmental factors [1,2]. In most instances, the cause of male infertility is not determined. This condition, known as idiopathic male infertility, is particularly challenging to diagnose and treat. Assisted Reproductive Technology (ART), particularly in vitro fertilization (IVF), is routinely used to manage these unexplained cases [3]. Despite the advances in reproductive medicine, however, the molecular mechanisms underlying idiopathic male infertility are poorly understood [3]. Recent studies have once again shifted focus to the molecular composition of sperm, and more importantly, the role of RNAs in male fertility potential [4].

It is now broadly acknowledged that spermatozoa convey not just genetic data in the form of DNA but also a diversity of RNA types, such as messenger RNAs (mRNAs) and small non-coding RNAs, including microRNAs (miRNAs), Piwi-interacting RNAs (piRNAs), and transfer RNA fragments, among others [[5], [6], [7], [8]]. These RNA molecules are thought to have important functions in the processes of spermogenesis, fertilization, and early embryogenesis [6,9]. The development of omics technologies such as transcriptomics, proteomics, metabolomics, and miRNomics has allowed further exploration of these RNA entities [[10], [11], [12]]. These strategies have identified promising biomarkers for sperm quality and reproductive potential. The biological roles and clinical significance of most of the small RNAs in human sperm are unknown. In particular, piRNAs have been shown to safeguard genome integrity during spermatogenesis by silencing transposable elements and regulating gene expression. These molecules remain in the mature sperm and suggest a possible role in fertilization and embryogenesis [6,9]. Similarly, miRNAs have been linked to the regulation of gene expression and epigenetic programming during sperm development [[6], [7], [8]]. Deregulated miRNA expression has been shown to influence DNA methylation patterns and chromatin remodeling, which are critical for sperm function and embryo quality [13]. Importantly, recent reports have also highlighted the potential of miRNAs and other small RNAs as valuable biomarkers for the prediction of pregnancy outcome, particularly in ART treatments such as IVF [14,15]. For instance, small non-coding RNAs like miRNAs were found to differentiate embryos with higher developmental and implantation potential very well, pointing to their usefulness as non-invasive biomarkers for the selection of embryos for ART. Similarly, research reported that miRNA overexpression profiles in pooled spent culture media and sperm are associated with embryo quality and pregnancy outcome [14,15]. Their findings suggest that some of the miRNAs secreted by pre-implantation embryos can also be utilized as biomarkers for embryo selection, once again highlighting their prospects as non-invasive predictors of fertility. Although prior reports have profiled small RNAs in single sperm and in enriched/bulk sperm cohorts, few studies have integrated small-RNA sequencing with downstream clinical IVF outcomes. Much of the earlier literature relies on bulk sperm populations, where residual somatic cells can confound RNA measurements and limit clinical applicability. To minimize such confounding, we combined standard purification with micromanipulation-based isolation of individual sperm, a routine IVF/ICSI approach that reduces non-sperm contamination and enables accurate assessment of sperm-specific RNA content [10]. They also provide the potential for analyzing rare or low-abundance RNA species with higher resolution. Nevertheless, the interconnections of small RNA expression with sperm quality and ART outcome remain largely unclear. It is essential to examine how specific RNAs are associated with conventional semen parameters, hormone levels, embryo development, and pregnancy rates. If such molecular signatures could be defined, it would lead to more precise diagnostic tests and strengthen personalized treatment regimens in male infertility.

The overall objective of the study was to assess the diagnostic utility of small RNAs, miRNAs and piRNAs, as related to sperm motility, morphology, and pregnancy outcomes. For this purpose, we examined selective sampling of single spermatozoa of male partners of women undergoing ART. Sperm were selected according to stringent morphological and motility parameters. We employed a micromanipulation instrument to obtain systematically three independent sperm samples from each participant. Each sample contained 1500 purified individual sperm cells. This stringent selection protocol reduced the risk of somatic cell contamination and aided in the assurance that the RNA expression patterns observed were inherent to the sperm cells. Total RNA was isolated from these purified sperm populations and was subjected to initial screening by small RNA sequencing (sRNA-seq) in a discovery cohort. The key miRNAs discovered by sequencing were subsequently confirmed in a second, larger cohort by quantitative real-time PCR (RT-qPCR) on unbiased sperm samples. In the investigation of both common and uncommon small RNAs in human sperm and their relation to traditional markers of fertility, this research seeks to advance the creation of precise RNA-based diagnostic methods for the assessment of male infertility and the prediction of the success of ART.

## 2 Materials and methods

### 2.1 Subjects of study

This study included 98 men, categorized into two cohorts. The first cohort (screening cohort) comprised 13 male partners from couples attending an infertility clinic. These men had not conceived after at least 12 months of unprotected intercourse and met the semen parameter criteria for male infertility as defined by the World Health Organization (WHO) 2010 guidelines. Specifically, infertility was defined when semen samples presented one or more of the following: sperm concentration <15 million/mL, total sperm number <39 million per ejaculate, progressive motility <32 %, normal morphology <4 %, or vitality <58 % live spermatozoa. The second cohort (validation cohort) included 85 male partners from couples seeking infertility treatment at a specialized center. They exhibited one or more of the following conditions, defined according to the WHO 2010 guidelines: low sperm count (oligozoospermia: sperm concentration <15 million/mL or total sperm number <39 million per ejaculate), reduced motility (asthenozoospermia: progressive motility <32 %), abnormal morphology (teratozoospermia: <4 % normal forms by strict criteria), or a combination of two or all three factors. These participants also had a history of infertility lasting at least 12 months and were actively undergoing treatment.

Male partners with systemic diseases were not included in our study. Eligibility for the study was based on failure to conceive after 12 months of unprotected intercourse. Clinical and semen characteristics for the first and second cohorts are summarized in Supplemental Table 1 and Supplemental Table 2, respectively.

Ethical approval was obtained from the Ethics Committees of Saarland Medical Association (Ha 195/11/updated June 2021 and NF. 52/25), Germany, following the Declaration of Helsinki principles. Additional approval was granted by the Institutional Review Board (IRB) of the Saarland Medical Association. Written informed consent was obtained from all participants.

### 2.2 Semen analysis and sperm collection

Following the initial semen analysis, samples were layered onto 45 %–90 % discontinuous PureSperm® gradients (Nidacon International, Cat. No. PS100-100). In some cases, an additional swim-up step was performed to further enrich for high-quality sperm. The processed sperm were then washed and resuspended in a minimal volume of medium for intracytoplasmic sperm injection (ICSI) and subsequent analysis.

**Fig. 1:**
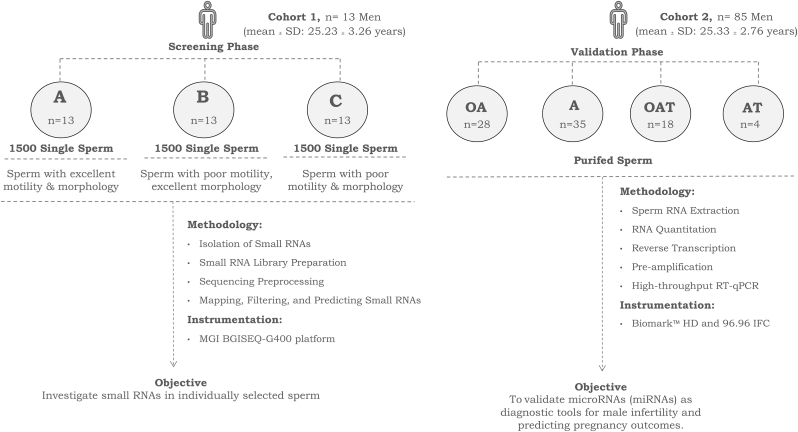
Flowchart of the Study Design: An overview of the study process, focusing on the analysis and validation of small RNAs in sperm.

Skilled embryologists used a micromanipulator (CooperSurgical) with a high-resolution camera (Nikon Corporation) to select sperm from the first cohort. Sperm were categorized based on motility and morphology according to **WHO 2010 criteria**, as illustrated in Fig. 1:

• **Group A (n** = **13):** Progressive motility ≥32 % and normal morphology ≥4 % (strict criteria, intact head, midpiece, and tail with no significant abnormalities); suitable for ICSI.
• **Group B (n** = **13):** No motility but normal morphology ≥4 %.
• **Group C (n** = **13):** No motility and abnormal morphology <4 %.

For sRNA-seq analyses, 1500 sperm cells were individually selected from each group (A, B, and C) for all 13 participants, resulting in 39 samples.

### 2.3 miRNA validation analysis

To validate the miRNA profiling results, an additional 85 samples were analyzed, collected from couples undergoing infertility treatment. The samples were purified and classified into the following categories, based on **WHO 2010 thresholds** (Fig. 1):

• **Asthenozoospermia (A, n** = **35):** Progressive motility <32 %.
• **Oligoasthenozoospermia (OA, n** = **28):** Sperm concentration <15 million/mL or total sperm number <39 million per ejaculate, combined with progressive motility <32 %.
• **Oligoasthenoteratozoospermia (OAT, n** = **18):** Sperm concentration <15 million/mL or total sperm number <39 million, progressive motility <32 %, and normal morphology <4 %.
• **Asthenoteratozoospermia (AT, n** = **4):** Progressive motility <32 %, combined with normal morphology <4 %.

Sample processing followed the same protocols as the sRNA-seq analysis to maintain consistency across experiments.

### 2.4 Isolation of small RNAs for small RNA sequencing

RNA was isolated from sperm samples using the miRNeasy Micro Kit (Qiagen). For the screening cohort, RNA was extracted from 39 samples comprising Group A (n = 13), Group B (n = 13), Group C (n = 13), and a control media sample (G-MOPS™, n = 1). The G-MOPS™ control media (Vitrolife), which was not exposed to sperm, was obtained from the same lot as the media used during sperm processing. Each sample was homogenized in 350 μL of QIAzol® Lysis Reagent (Qiagen) supplemented with Dithiothreitol (DTT) (80 mmol/L, Sigma-Aldrich) and incubated for 60 min at room temperature with gentle agitation. The isolation process was then performed using the QIAcube™ robot (Qiagen) in accordance with the manufacturer's protocol. RNA was eluted in 14 μL of RNase-free water for each sample. Due to the minimal RNA quantity, concentrations could not be determined using a Nanodrop or Qubit™ RNA High Sensitivity (HS) Assay Kit with the Qubit™ 4 Fluorometer (Thermo Fisher Scientific). However, the presence of RNA, including small RNAs, was confirmed using the RNA 6000 Pico Kit on the Agilent 2100 Bioanalyzer™ (Agilent Technologies), which displayed characteristic band patterns indicative of small RNA species. The miRNeasy Micro Kit was also used to isolate RNA from an additional 85 sperm samples from the validation cohort to validate findings from sRNA-seq analyses.

### 2.5 Small RNA libraries preparation and sequencing

The MGIEasy Small RNA Library Prep Kit (MGI Technologies) was employed to construct small RNA libraries for sperm samples and G-MOPS™ (n = 39 samples; Group A: n = 13, Group B: n = 13, Group C: n = 13, and G-MOPS™: n = 1). To minimize technical variability, all 40 screening cohort samples were processed in a fully standardized workflow. RNA extraction, library preparation, and sequencing were performed using automated platforms in a single batch, with libraries pooled and sequenced together on one flow cell using reagents from the same lot. This approach ensured consistency and eliminated inter-batch effects.

To eliminate any potential contamination from residual RNAs present in the media, samples were thoroughly washed multiple times. As a control, the media not exposed to sperm was also sequenced to confirm that any detected RNA signatures originated solely from the media itself. Following elution, approximately 11 μL of RNA was further concentrated to 6 μL using a SpeedVac™ (Thermo Fisher Scientific). RNA library preparation was then performed according to the manufacturer's instructions on the MGISP-960 automated library preparation workstation (MGI Technologies), utilizing a 6 μL protocol.

The process began with ligating adapter sequences to the 3′ and 5′ ends of the RNA, followed by reverse transcription to generate complementary DNA (cDNA) using primers containing unique barcodes. The cDNA was then amplified via PCR using 18 cycles in a 100 μL reaction. Size selection of PCR products within the 100–120 base pair range was achieved using AMPure® XP beads (Beckman Coulter). The purified PCR product size was verified using the DNA 1000 Kit on the Agilent 2100 Bioanalyzer (Agilent Technologies), and quantification was performed with the Qubit™ 1X dsDNA HS Assay Kit on the Qubit™ 4 Fluorometer (Thermo Fisher Scientific). Subsequently, single-strand circularization of the DNA library was conducted as per the manufacturer's protocol. Quantification of the single-stranded circularized DNA was performed using the Qubit™ ssDNA Kit on the Qubit™ 4 Fluorometer. Finally, the resulting library was sequenced using the MGI BGISEQ-G400 platform with the High-throughput Sequencing Set (Small RNA), following the manufacturer's guidelines.

### 2.6 Processing, mapping, filtering, and prediction of small RNA sequences

The FASTQ files containing small RNA sequencing data were subjected to a multi-step processing workflow. Initially, the raw data underwent a quality check, followed by adapter trimming using Cutadapt [16], a specialized tool for removing unwanted adapter sequences from high-throughput sequencing reads. The quality of the trimmed data was re-evaluated using FastQC. Subsequently, the trimmed reads were aligned to the human reference genome (hg38 assembly) using Bowtie [17]. To ensure comprehensive annotation, the miRNA database from miRBase and the non-coding RNA database from RNAcentral were updated with the latest collections of published miRNA and non-coding RNA sequences. The aligned reads were processed to calculate the abundance of various small RNA types. Quantification of miRNA and other small RNAs was performed using DESeq2 [18], and the identification of differentially expressed small RNAs was further confirmed through edgeR [19]. Finally, downstream analyses, including data visualization, were carried out using R software (v4.2.1, R Core Team), enabling a detailed exploration of the differentially expressed small RNA profiles.

### 2.7 Reverse transcription and quantitative real-time PCR (RT-qPCR) of miRNAs and mRNAs

The expression levels of differentially expressed miRNAs were quantified using RT-qPCR on the Biomark HD System (Fluidigm). RNA quality was assessed, and a total of 85 samples were validated. Complementary DNA (cDNA) was synthesized in 12 μL reactions by reverse-transcribing 75 ng of total RNA using the TaqMan™ MicroRNA Reverse Transcription Kit (Thermo Fisher Scientific) following the manufacturer's protocol. The cDNA (2.5 μL) underwent pre-amplification in a 25 μL reaction containing 12.5 μL of TaqMan™ PreAmp Master Mix (2X) and 3.75 μL of a PreAmp Primers Pool (0.2X). The pre-amplified cDNA was then analyzed using RT-qPCR on a 96.96 Dynamic Array™ IFC for Gene Expression arrays (Fluidigm) with TaqMan™ MicroRNA Assays (Thermo Fisher Scientific) designed for each differentially expressed miRNA.

The Biomark HD instrument was used for miRNA quantification and detection, following specific thermal cycling protocols recommended by the manufacturer (Fluidigm Corporation). For miRNA normalization, RNU6B, miR-30a-5p, and miR-100-5p were selected as reference endogenous controls due to their minimal coefficient of variation (CV) between samples [10,20,21]. The CV, representing the ratio of the standard deviation to the mean, was used to identify miRNAs with the least variability among those considered for normalization. As a sensitivity analysis, we also applied a global normalization approach (averaging a broader set of stably expressed miRNAs); effect sizes and significance calls were concordant with the primary analysis. To minimize reliance on a single reference and increase robustness, target miRNA expression was normalized to a combined multi-miRNA reference, calculated as the geometric mean of the three most stable controls: RNU6B (CV = 0.136), miR-30a-5p (CV = 0.141), and miR-100-5p (CV = 0.155). As a sensitivity analysis, we also applied a global normalization approach, averaging a broader set of stably expressed miRNAs. The resulting effect sizes and significance calls were consistent with those from the primary analysis. Controls, including No-Template Control (NTC) and RT-negative controls, were included in every run to ensure data accuracy and reliability.

### 2.8 Statistical and bioinformatics analysis

All statistical analyses were performed in R software (v4.2.1, R Core Team). For small RNA-seq data, raw counts were summarized by RNA class, and relative abundances were expressed as proportions of total annotated reads. Differential expression of sRNA-seq data was assessed using DESeq2 and edgeR to ensure validation and reproducibility; DESeq2 was used for final data interpretation owing to its established robustness and reliability. Small RNAs were retained for downstream analyses if they exhibited ≥10 raw read counts in at least 10 of 13 samples within each group (A, B, C). Compositional profiles were visualized with 100 % stacked bar charts, while differential expression results obtained from external pipelines were displayed using volcano plots (thresholds: log_2_ FC = ±1, adjusted q = 0.05). Overlap of differentially expressed features across pairwise group comparisons was illustrated with Venn diagrams. For clinical and sperm parameters, distributional assumptions were assessed using the Shapiro–Wilk test. Two-group comparisons (e.g., β-hCG outcome, embryo grade, live-birth outcome) were performed with the Mann–Whitney *U* test. Three-group comparisons were analyzed using one-way ANOVA with Tukey's HSD post hoc test when assumptions were met; otherwise, the Kruskal–Wallis test with Dunn's pairwise comparisons was applied. Associations between small RNA expression and clinical parameters were evaluated with Spearman's rank correlation, and scatterplots were used to visualize correlation strength and direction. For RT-qPCR validation experiments (n = 85), expression levels were calculated using the ΔCt method. Coefficients of variation were computed for each feature, and associations with sperm and clinical traits were tested using Spearman's correlation. Group differences were analyzed with the Mann–Whitney *U* test for two groups or the Kruskal–Wallis test with Dunn's post hoc procedure for more than two groups. To account for multiple testing, Benjamini–Hochberg false discovery rate (FDR) adjustment was applied throughout; adjusted q < 0.05q < 0.05q < 0.05 was considered statistically significant [22]. Finally, diagnostic performance for predicting reproductive outcomes was assessed using receiver operating characteristic (ROC) analysis. Logistic regression models were fitted for individual features as well as combined biomarker panels, and area under the curve (AUC) with 95 % confidence intervals was calculated. ROC curves were plotted to visualize sensitivity and specificity, with the diagonal line included as a reference for random classification.

## 3 Results

### 3.1 Evaluation of the demographic, hormonal, and clinical characteristics (screening cohort)

Correlation analysis was performed to examine the relationships among basic sperm parameters, embryo quality, and serum levels of β-hCG, FSH, and LH in a screening cohort comprising 13 male partners (age 20–30 years; mean ± SD: 25.23 ± 3.39) and their female partners (age 20–30 years; mean ± SD: 25.39 ± 3.17). (Supplemental Table 1). In spite of the small sample size, an appreciable number of significant findings were observed. In grading the embryos, increased sperm count, and progressive motility have been associated with embryos of better quality. In particular, men with embryos graded G1 or G1/G2 had sperm counts of 40–65 million/mL, with progressive motility percentages of 11–30 %. In contrast, individuals who had unfavorable results such as 'No Oocyte Retrieval' or 'No Cleavage' had reduced sperm counts, with values decreasing to as low as 18 to 20 million/mL. Normal sperm morphology was also associated with embryo quality; presentations with good embryo grades also had morphology percentages ranging from 7 to 12 %, whereas lower morphology percentages (3–5 %) were also associated with poor outcomes. When considering β-hCG levels, a marker of successful pregnancy, successful outcomes were observed over a broad range of sperm counts (14–55 million/mL), suggesting no direct association between sperm count and successful pregnancy. However, progressive motility was more strongly associated with β-hCG positivity, with motility ranges of 10–30 % being generally observed for positive samples (Supplemental Table 1).

Conversely, poor β-hCG outcomes were more closely linked with decreased or absent progressive motility. Additionally, sperm morphology also revealed a link to successful pregnancies, whereby positive β-hCG outcomes were correlated with morphology percentages ranging from 4 % to 12 %. Furthermore, hormonal profiles also revealed correlations with pregnancy outcomes. In positive β-hCG, FSH and LH values tended to be lower (2.2–5.6 ng/mL and 3.5–8.7 ng/mL, respectively), while abnormal FSH and LH values were more usually present in the negative outcomes (Supplemental Table 1).

### 3.2 Processing and annotation of small RNA-seq reads

**Fig. 2:**
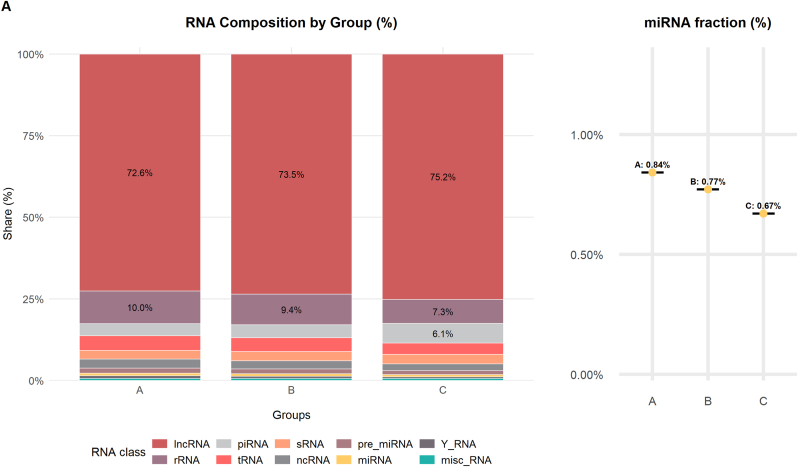

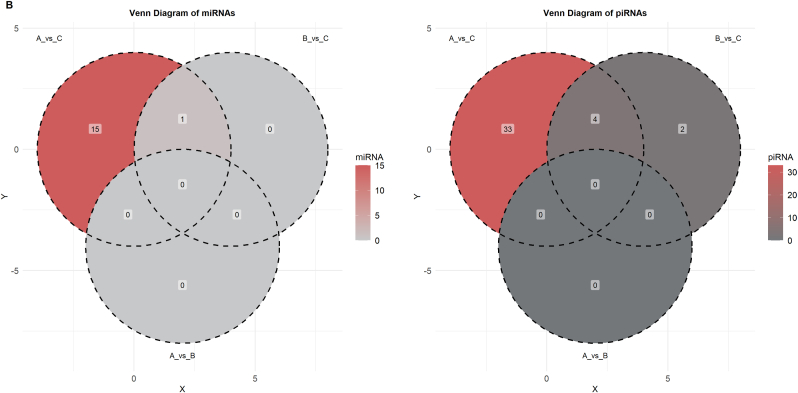

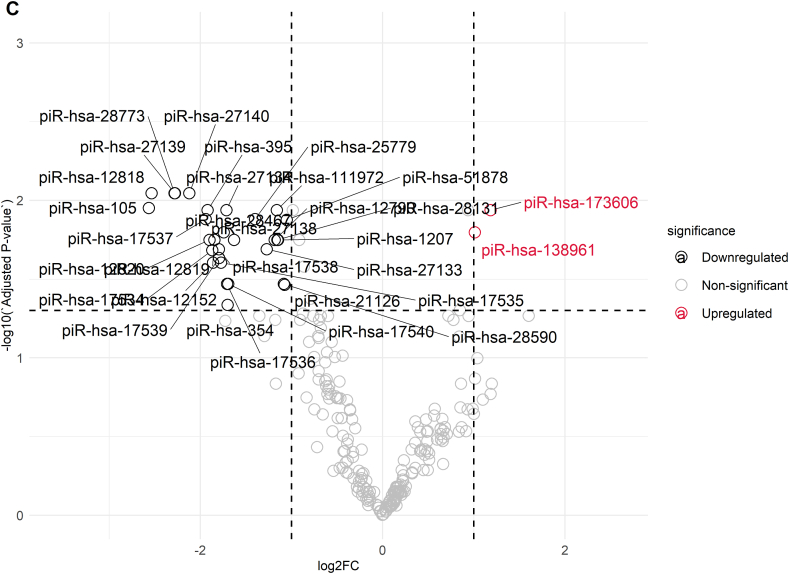

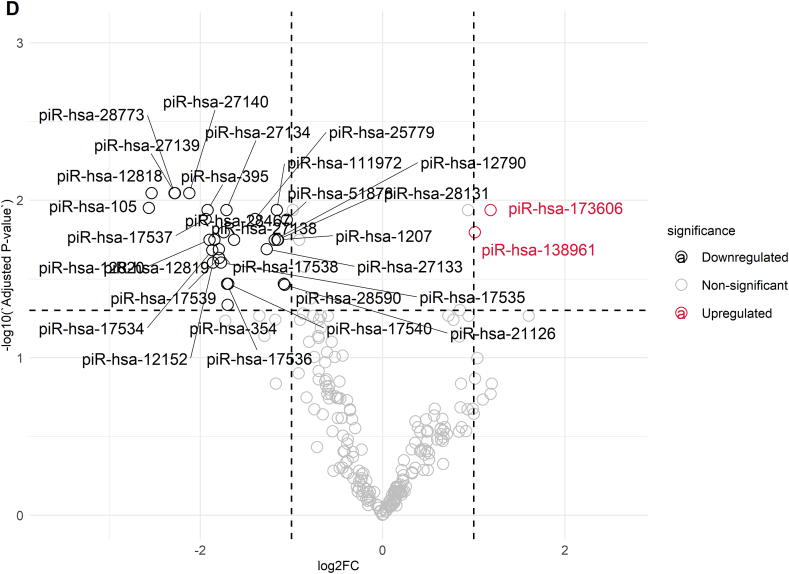

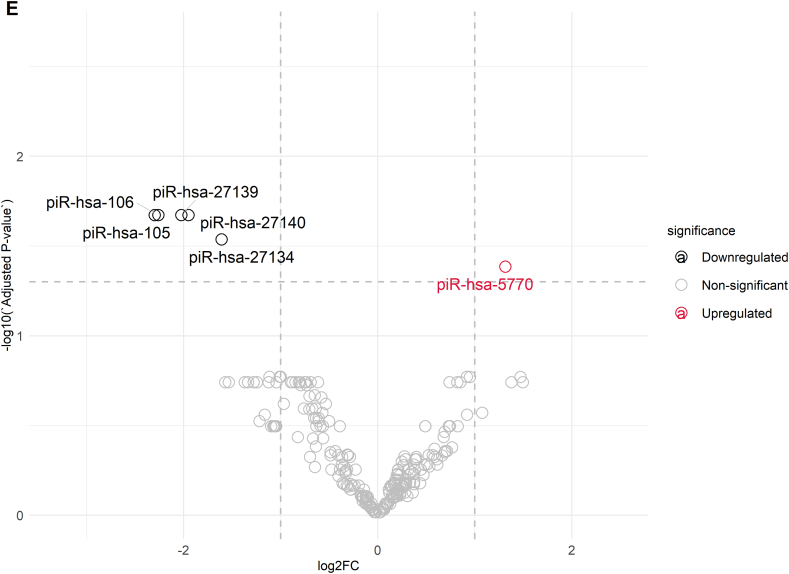
Analyses of Small RNA Biotypes in Individually Selected Sperm Samples. A) This shows the distribution of common small RNA biotypes in the selected sperm samples, expressed as percentages. B) A Venn diagram illustrates the overlap of miRNAs and piRNAs detected across Groups A, B, and C of the selected sperm samples. C) A volcano plot depicts the differential expression of miRNAs between Group A and Group C. D) Another volcano plot shows the differential expression of piRNAs between Group A and Group C. E) A volcano plot demonstrates the differential expression of piRNAs between Group B and Group C.

The analysis and annotation of small RNA sequencing reads included the assessment of 39 various libraries, which belonged to three groups: Group A, Group B, and Group C, with 13 samples in each group. The first sequencing yielded approximately 483.5 million reads. After trimming adapter sequences and low-quality bases, approximately 82 million reads were mapped to the reference genome (Supplemental Table 3). The percentages of alignment within the groups were 14.15 % ± 4.41 % for Group A, 15.75 % ± 5.15 % for Group B, and 15.95 % ± 6.25 % for Group C. Small RNAs accounted for 4.14 % of the reads aligned, with 3.35 % in Group A, 3.84 % in Group C, and 3.35 % in Group B. Fig. 2A illustrates the distribution of RNA classes among the mapped sequences. The most prevalent type of RNA in the study was long non-coding RNA (lncRNA) at 72.69 % ± 1.04 %. The other RNAs present were ribosomal RNA (rRNA) at 8.79 % ± 1.12 %, transfer RNA (tRNA) at 3.97 % ± 0.46 %, and PIWI-interacting RNA (piRNA) at 4.64 % ± 0.99 %. The following types of RNA had lower abundances: non-coding RNA (ncRNA) at 2.42 % ± 0.31 %, small RNA (sRNA) at 2.75 % ± 0.13 %, precursor microRNA (pre-miRNA) at 1.37 % ± 0.11 %, Y RNA at 0.68 % ± 0.11 %, microRNA (miRNA) at 0.70 % ± 0.06 %, miscellaneous RNA (misc_RNA) at 0.54 % ± 0.01 %, signal recognition particle RNA (SRP_RNA) at 0.46 % ± 0.04 %, small nuclear RNA (snRNA) at 0.57 % ± 0.04 %, small nucleolar RNA (snoRNA) at 0.26 % ± 0.04 %, circular RNA (circRNA) at 0.07 % ± 0.01 %, antisense RNA at 0.03 % ± 0.01 %, small Cajal body-specific RNA (scaRNA) at 0.02 % ± 0.01 %, and other small RNAs at 0.06 % ± 0.01 %. In addition to the main classes of RNA, some regulatory RNAs, such as piRNAs and miRNAs, were present at varying concentrations among the groups, as shown in Supplemental Table 3. Notably, the piRNA content ranged from 3.72 % in Group A to 5.96 % in Group C.

Contrary to this, miRNAs, which have been implicated in the regulation of gene expression, were comparatively less abundant across the groups, decreasing from 0.83 % in Group A to 0.66 % in Group C. These findings point to the heterogeneous composition of small RNA species in the libraries being investigated, with an interest in the very dominant and the less dominant forms of RNA, particularly in light of the regulatory role of piRNAs and miRNAs.

### 3.3 Differentially expressed miRNAs and piRNAs

Differential expression analysis observed the presence of differential expression profiles of some small RNAs among the three different groups (A, B, and C) of sperm samples, which were categorized on the basis of motility and morphological characteristics. Group A included sperm with higher motility and morphology, while Group C included sperm with zero motility and with aberrant morphological characteristics.

As indicated by Fig. 2B, the Venn diagram presents the comparative summary of the remarkable miRNAs and piRNAs discovered in each group. For miRNA analysis, an overall differential comparison of Group A versus Group C revealed 16 miRNAs with significant difference in expression (adjusted p < 0.05). In the processed data set, 14 miRNAs were significantly downregulated, and 2 miRNAs showed upregulation in Group A when compared with Group C. Most downregulated miRNAs were hsa-miR-223-3p (log2FC = −5.19), hsa-miR-424-5p (log2FC = −2.48), hsa-miR-199a-5p (log2FC = −2.59), and hsa-miR-199b-5p (log2FC = −2.59) and hsa-miR-15a-5p (log2FC = −1.37). The remaining miRNAs that were downregulated were hsa-miR-15b-5p (log2FC = −2.03), hsa-miR-19a-5p (log2FC = −1.94), hsa-miR-106b-5p (log2FC = −0.94), hsa-miR-449b-5p (log2FC = −2.10), hsa-miR-335-5p (log2FC = −1.85), hsa-miR-449a (log2FC = −2.09), hsa-miR-20a-5p (log2FC = −0.76), hsa-miR-16-5p (log2FC = −1.12), and hsa-miR-146a-5p (log2FC = −0.85). In contrast, the two miRNAs that were upregulated were hsa-miR-149-5p (log2FC = 1.38) and hsa-miR-99a-5p (log2FC = 0.74) (Fig. 2C). In contrast, Group B, which was defined by sperm with normal morphology but impaired motility, was discovered to have a unique pattern of expression. In comparison with Group C only, only a single miRNA, hsa-miR-223-3p, was significantly differentially expressed (log2FC = −3.40, p = 1.59 × 10^−2^) (Fig. 2B). Importantly, no miRNA expression showed statistically significant differences between Groups A and B, implying molecular similarities with functional differences in sperm.

In the piRNA analysis, comparative differential expression between Groups A and C identified 37 significantly differentially expressed piRNAs (adjusted p < 0.05) (Fig. 2D). In this group, 33 piRNAs were downregulated, and 4 piRNAs were upregulated in Group A as compared to Group C. The most downregulated piRNA was piR-105 (log2FC = −2.57), followed by piR-12818 (log2FC = −2.54), piR-28773 (log2FC = −2.28), piR-27139 (log2FC = −2.28), and piR-27140 (log2FC = −2.12). In contrast, four piRNAs had higher expression in Group A than in Group C, such as piR-173606 (log2FC = 1.18) and piR-138961 (log2FC = 1.01). Six piRNAs with differential expression were detected by comparison of Group B and Group C, as indicated in Fig. 2E (adjusted p < 0.05), among which five had decreased expression and one had increased expression. Among downregulated piRNAs, the most decreased was piR-105 (log2FC = −2.30), followed by piR-106 (log2FC = −2.26) and piR-27139 (log2FC = −2.02). The only piRNA that showed upregulation in this study was piR-5770 (log2FC = 1.31). In accordance with the miRNA analysis, there were no significant differences in piRNA expression between Groups A and B.

### 3.4 Correlation analysis of differentially expressed miRNAs and sperm parameters

A Spearman's correlation analysis was carried out to ascertain if there were correlations between read counts of variously expressed miRNAs and some sperm quality parameters, viz., Progressive Motility (PM), Immobility (IM), and morphology (M). Correlations were found to be significant only in Group A, but Groups B and C did not show any correlations. There was a remarkable trend with implications of considerable negative correlations between miRNA expression levels and PM, along with M, in contrast to positive correlations with IM (p < 0.05), as indicated by Supplemental Fig. 1. For instance, hsa-miR-15a-5p was significantly negatively correlated with PM (r = −0.575) and M (r = −0.558) but positively correlated with IM (r = 0.725). Likewise, hsa-miR-15b-5p and hsa-miR-16-5p were negatively correlated with PM (r = −0.707; r = −0.670) and positively correlated with IM (r = 0.727 for both). hsa-miR-19a-5p was also negatively correlated with PM (r = −0.559) and positively correlated with IM (r = 0.686). hsa-miR-20a-5p had inverse relationships with PM (r = −0.553), and positive and inverse relationships with IM (r = 0.667) and M (r = −0.599).

Additionally, hsa-miR-199a-5p and hsa-miR-199b-5p also manifested the same tendencies, with high negative correlations with PM (r = −0.575). hsa-miR-106b-5p was significantly negatively correlated with M (r = −0.556), whereas it was positively correlated with IM (r = 0.690). Also, hsa-miR-149-5p presented significant correlations with IM (r = 0.584). In addition, hsa-miR-99a-5p also presented a significant correlation with IM (r = 0.661). The findings indicate a possible regulatory role of certain miRNAs in relation to sperm motility and morphology, especially in Group A, where there were significant correlation patterns observed.

### 3.5 Correlation analysis of differentially expressed piRNAs and sperm parameters

Based on the knowledge gained using the miRNA analysis, the same approach was used in investigating differentially expressed piRNAs along with sperm characteristics. Similar to the findings for miRNAs, correlations were only significant in Group A, with no correlations in Groups B and C, as shown in Supplemental Fig. 2. In Group A, negative correlations were seen for PM and M, and positive correlations for IM sperm. In particular, piR-28773, piR-17540, and piR-25779 were significantly negatively correlated with PM (r = −0.569, r = −0.718, r = −0.615, respectively). Moreover, other piRNAs, including piR-28467, piR-12819, piR-12790, and piR-17534, were found to have significant negative correlations with PM. In IM, piRNAs such as piR-57648 (r = 0.81), piR-111972 (r = 0.68), piR-115220 (r = 0.672), piR-173606 (r = 0.603), and piR-51878, piR-138961, piR-675641, piR-256220, and piR-12423 had significant positive correlations. Regarding sperm M, different correlations were found; for instance, piR-111972 (r = −0.564) and piR-57648 (r = −0.638) had significant negative correlations. These findings also suggest the potential regulatory role of piRNAs in sperm activity, with correlation patterns differing in Group A.

### 3.6 Confirmation of miRNAs using a single real-time RT-qPCR analysis

#### 3.6.1 Validation cohort evaluation of demographic, hormonal, and clinical features

We performed correlation analysis to compare the relationships between sperm parameters, embryo quality, and serum levels of β-hCG, FSH, and LH in 85 couples receiving infertility treatment. The mean age of the male partners was 25.52 ± 2.81 years (mean ± SD), as presented in Supplemental Table 2. According to spermiogram results, men were diagnosed with various sperm status, such as OA, A, OAT, and AT.

**Fig. 3:**
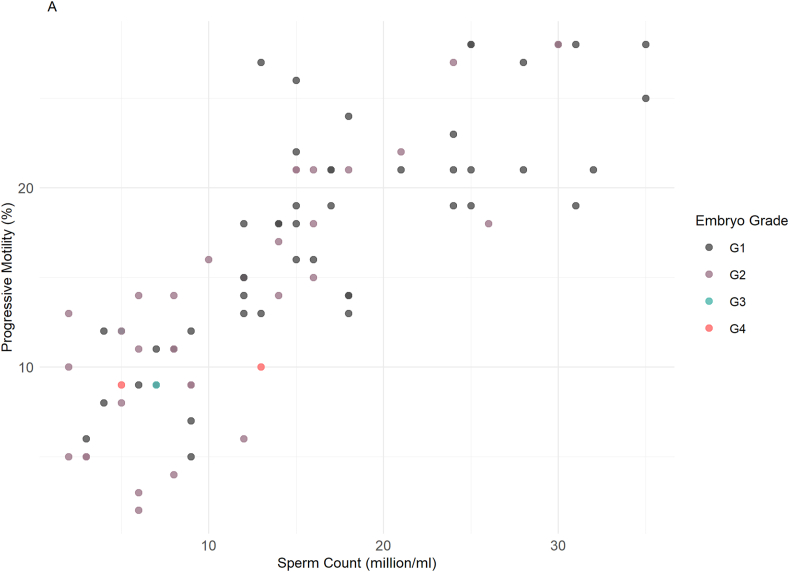

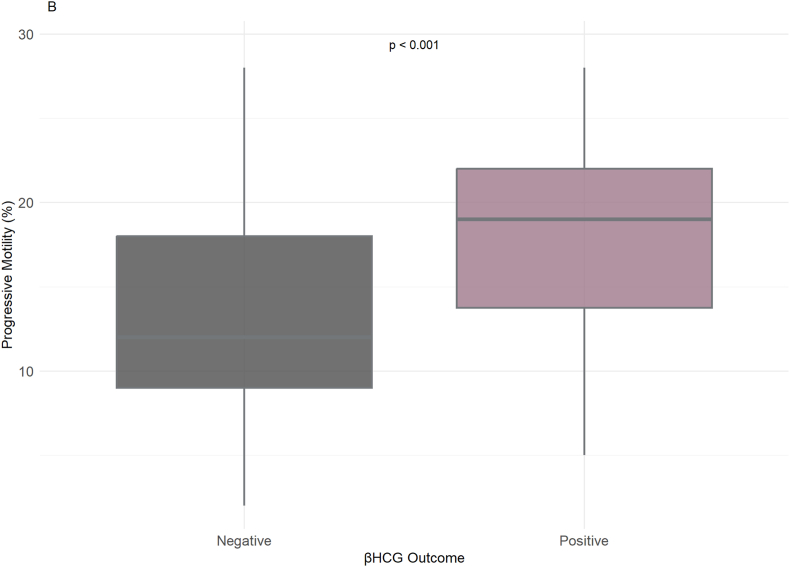

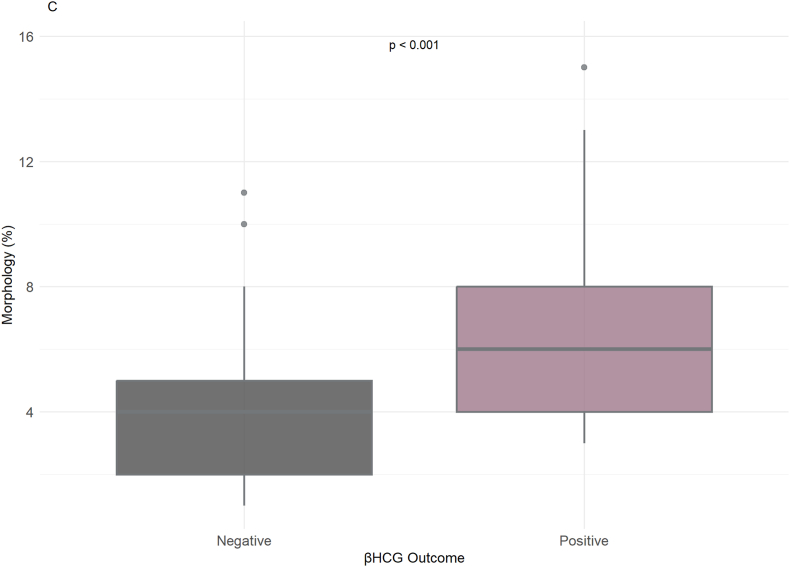

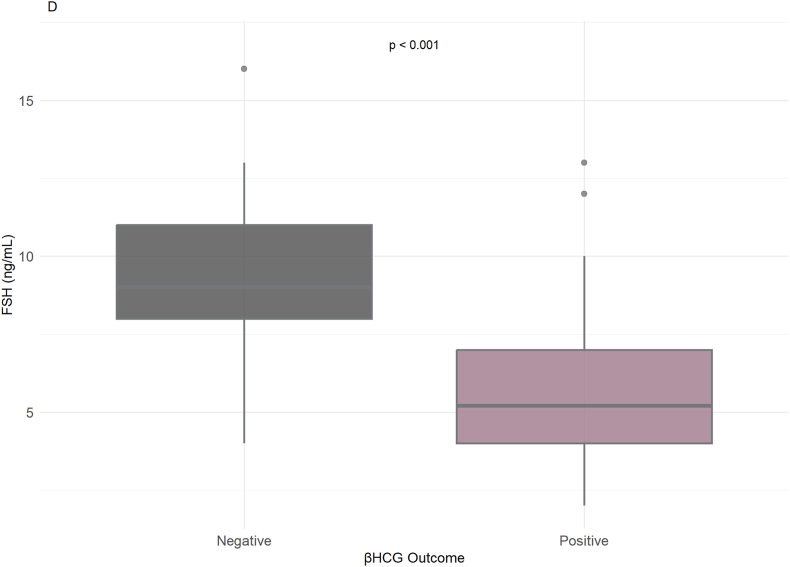

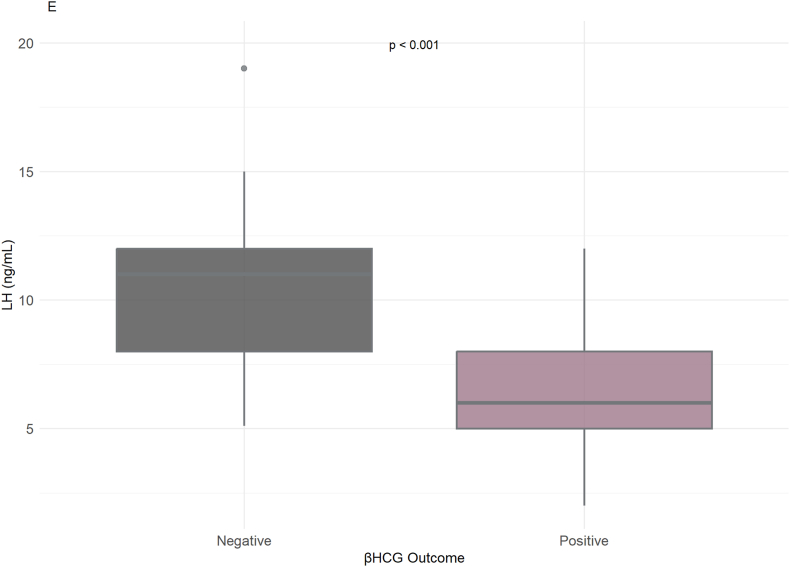

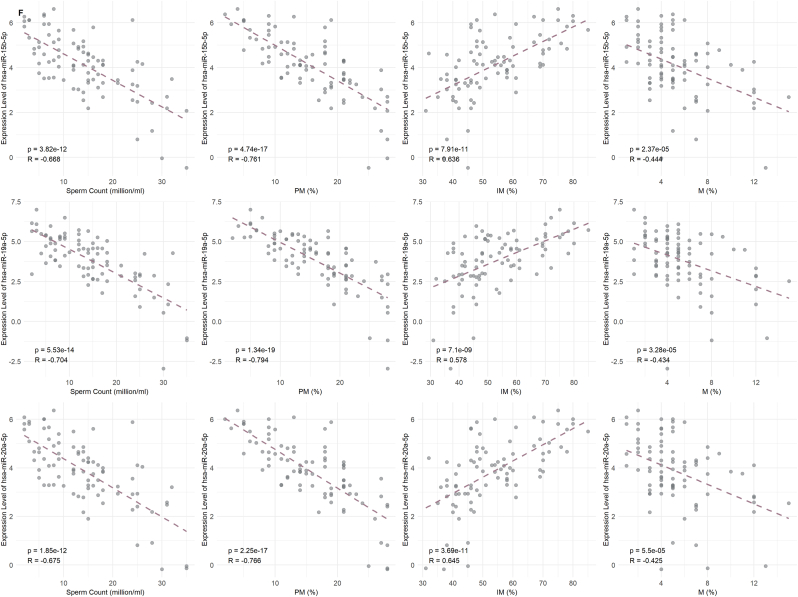
Correlation of Sperm and Clinical Parameters with miRNA Expression Levels. A) This illustrates the relationship between progressive motility (%) and sperm count (million/ml) across different embryo grades (G1-G4). B) A comparison of β-hCG outcomes (positive vs. negative) based on sperm count (million/ml). C) The relationship between LH levels (ng/ml) and β-hCG outcomes (positive vs. negative). D) This shows the relationship between FSH levels (mIU/ml) and β-hCG outcomes (positive vs. negative). E) Differences in progressive motility (%) between positive and negative β-hCG outcomes. F) Spearman correlation plots display the relationship between the expression levels of three miRNAs and various sperm parameters (sperm count, motility, morphology). The corresponding p-values and correlation coefficients (r) are noted for each plot. We identified statistical significance using adjusted p-value threshold of <0.05.

As shown in Fig. 3A, embryo grading demonstrated varying correlations with sperm count and progressive motility (PM). Fig. 3A illustrates the relationship between sperm count (× 10^6^/mL) and progressive motility (%), stratified by embryo quality grades (G1–G4). Among samples associated with G1 embryos, Spearman's correlation coefficient (r) was 0.800 (p < 0.0001, n = 48), indicating a strong and statistically significant association. Similarly, for G2 embryos, the correlation remained high (r = 0.806, p < 0.0001, n = 33). These findings suggest that, within both groups, higher sperm counts are consistently associated with greater motility. Sperm concentrations linked to G1 embryos ranged from 2 to 35 × 10^6^/mL, with progressive motility between 2 % and 28 %. In contrast, G2-associated samples showed intermediate sperm concentrations and motility, typically falling between values observed in G1 and G3 groups. Supplementary Table 2 presents a boxplot of normal sperm morphology (%) across embryo grades. For G1 embryos, morphology values clustered between 6 % and 8 %, while G2 morphology remained consistently near 7 %. Pairwise comparisons revealed a statistically significant difference in morphology between G1 and G2 groups (p = 0.0017). However, due to the limited sample sizes in the G3 and G4 categories, statistical analyses were not performed for these groups to avoid misleading conclusions. Morphology values for G3 embryos were more variable and frequently dropped below 6 %. Embryos classified as G4 were associated with the lowest sperm concentrations (as low as 2 × 10^6^/mL), reduced motility (2 %–10 %), and morphology values often below 5 %, with a wider distribution (Supplemental Table 2).

Regarding the β-hCG outcome, an indicator of pregnancy success, sperm morphology (%) appeared to differ between groups (ρ < 0.001), with higher morphology percentages (approximately 4 %–12 %) observed in the positive β-hCG outcome group compared to the negative group (Fig. 3B). Although sperm count (million/mL) showed a broad range in both groups, its distribution did not strongly distinguish between outcomes. In terms of progressive motility (%), men with higher motility values (ranging from 10 % to over 20 %) were more likely to have a positive β-hCG outcome (ρ < 0.001), suggesting a stronger association between motility and successful pregnancy (Fig. 3C).

Hormonal analysis showed that LH (ng/mL) levels were generally lower in the positive β-hCG outcome group, while higher LH concentrations were observed in the negative group (ρ < 0.001) (Fig. 3D). Similarly, FSH (ng/mL) levels were elevated in men with a negative β-hCG outcome, and lower FSH values were associated with pregnancy success (ρ < 0.001) (Fig. 3E). These findings suggest an inverse relationship between FSH/LH concentrations and β-hCG positivity.

#### 3.6.2 Age and sperm condition-independent miRNA expression analysis

Since the expression levels of miRNAs are strongly influenced by age and sex, we aimed to match the included samples with those used in the sRNA-seq analysis. After obtaining the Ct values for each miRNA, we assessed the effect of age on miRNA expression levels. As shown in Supplemental Fig. 3, correlation analysis revealed no significant association between miRNA expression levels and age, for all tested miRNAs (r < 0.1), indicating that age did not have a substantial impact on miRNA expression in this cohort.

To further quantify differences in miRNA expression across the four sperm classifications, computational and statistical methods were applied, including the Kruskal-Wallis test followed by multiple comparison corrections. The analysis revealed significant differences in miRNA expression levels among the groups, as illustrated in Supplemental Fig. 4. Notably, hsa-miR-146a-5p (adjusted p = 4.35 × 10^−6^) and hsa-miR-19a-5p (adjusted p = 4.35 × 10^−6^) exhibited the most pronounced differences, highlighting their potential as biomarkers for differentiating sperm conditions. Other miRNAs, such as hsa-miR-449b-5p (adjusted p = 9.64 × 10^−6^) and hsa-miR-199b-5p (adjusted p = 2.14 × 10^−5^), also displayed notable differences across the groups. Additionally, hsa-miR-15b-5p and hsa-miR-20a-5p demonstrated significantly higher expression levels (adjusted p = 2.97 × 10^−5^). The least significant miRNA identified was hsa-miR-99a-5p, with an adjusted p-value of 1.35 × 10^−3^ (Supplemental Fig. 4).

#### 3.6.3 Expression analysis of age and sperm condition-independent miRNA

Since miRNA expression levels are significantly influenced by age and sex, we attempted to match the samples included with the samples of sRNA-seq analysis. After determining the Ct value of each miRNA, we examined the correlation between age and miRNAs expression level. As indicated in Supplemental Fig. 3, the correlation analysis indicated that there was no substantial relationship between miRNA expression level and age for all miRNAs studied (r < 0.1), which suggested that age had no profound influence on miRNA expression in this cohort. In order to study miRNA expression differences among the four categories of sperm classification, various computational and statistical approaches were employed, including the Kruskal-Wallis test with subsequent multiple comparisons corrections. The comparison detected miRNA expression levels that differed significantly between the groups, as indicated in Supplemental Fig. 4. Specifically, hsa-miR-146a-5p (adjusted p = 4.35 × 10^−6^) and hsa-miR-19a-5p (adjusted p = 4.35 × 10^−6^) presented the most striking differences, indicating their prospects for application as biomarkers for the determination of sperm conditions. The remaining miRNAs, including hsa-miR-449b-5p (adjusted p = 9.64 × 10^−6^) and hsa-miR-199b-5p (adjusted p = 2.14 × 10^−5^), showed significant group differences. Moreover, hsa-miR-15b-5p and hsa-miR-20a-5p were found to be statistically significantly more expressed (adjusted p = 2.97 × 10^−5^). The least significant miRNA found was hsa-miR-99a-5p, with an adjusted p-value of 1.35 × 10^−3^ (Supplemental Fig. 4).

#### 3.6.4 miRNA expression level correlation with basic semen parameters

**Fig. 4:**
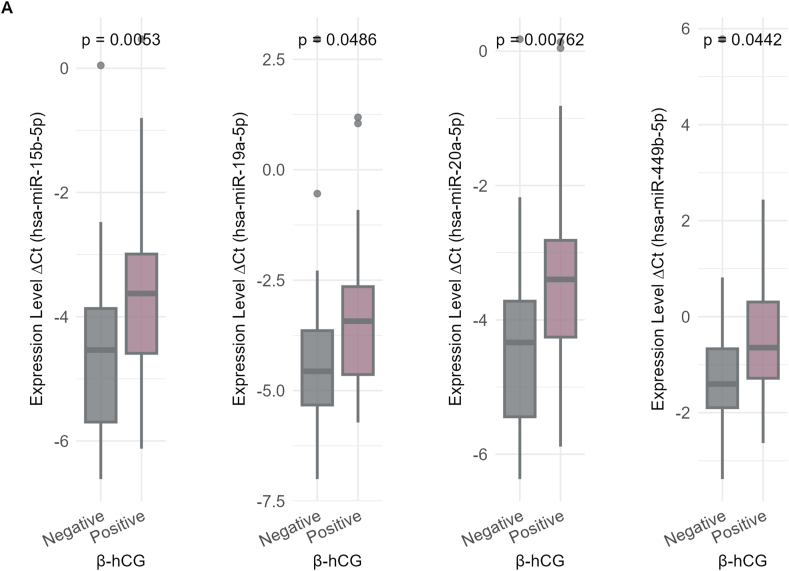

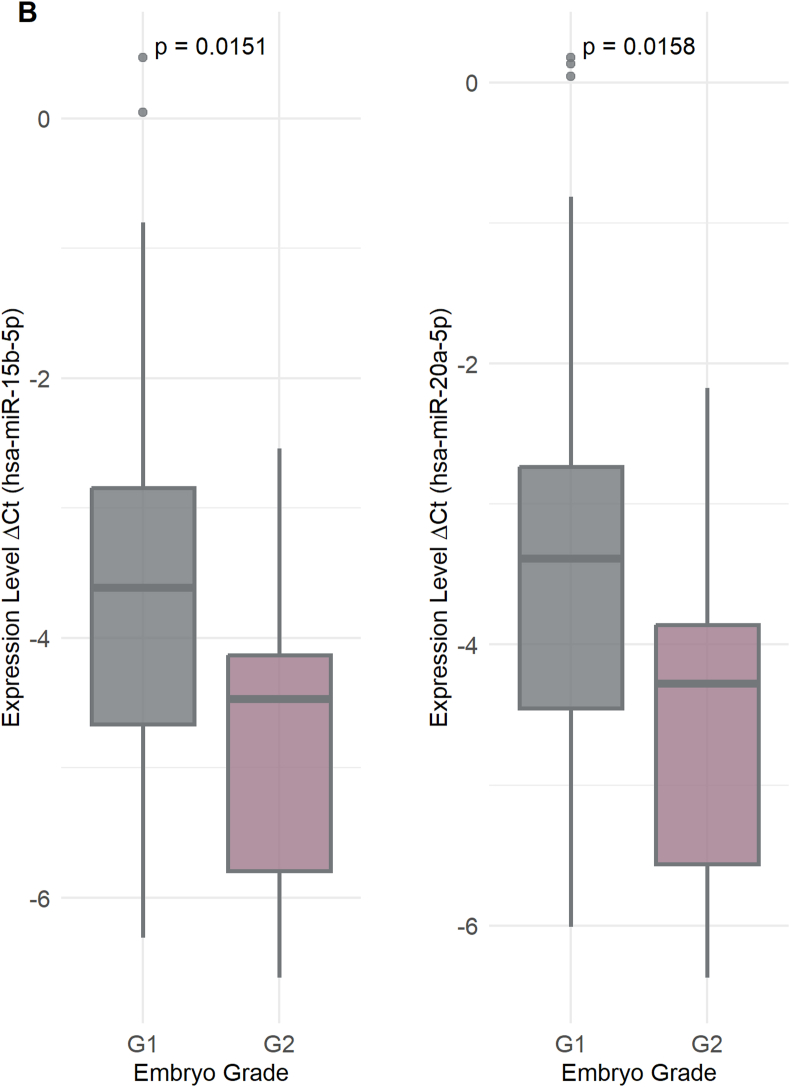

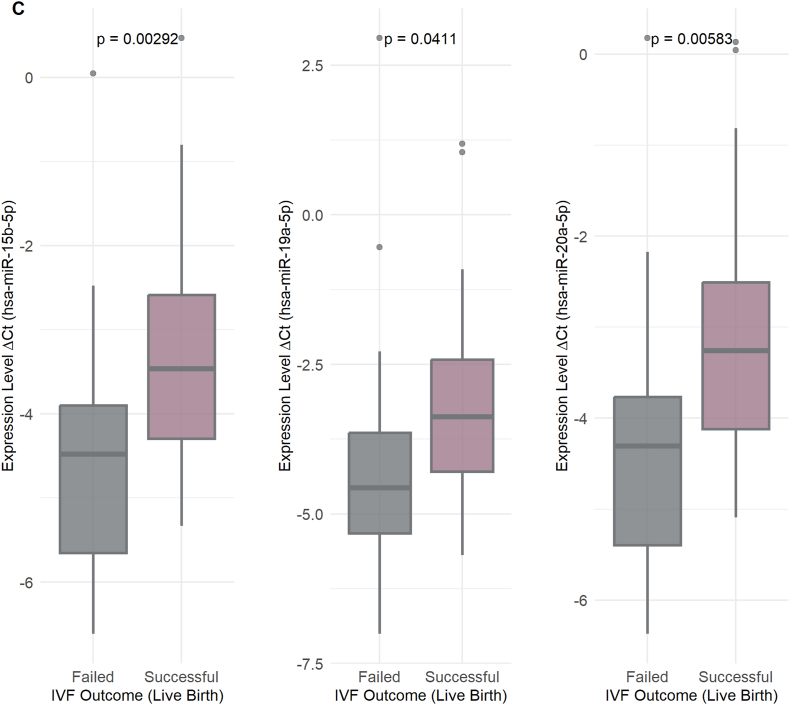

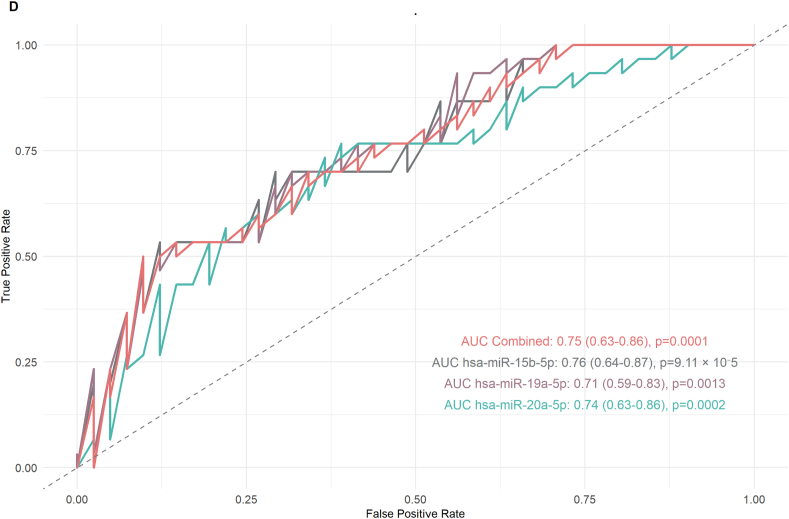
Association of miRNA Expression with Clinical Outcomes. A) This shows differentially expressed miRNAs between negative and positive β-hCG outcomes. B) Differentially expressed miRNAs across different embryo grades (G1 and G2). C) Differentially expressed miRNAs based on successful vs. failed IVF outcomes (live birth). D) Receiver Operating Characteristic (ROC) curves illustrate the diagnostic performance of three miRNAs, with their corresponding Area Under the Curve (AUC) values, p-values, and 95 % confidence intervals listed. Statistical significance was determined using adjusted p-value threshold of <0.05.

For analysis of the association between miRNA expression and basic semen parameters, namely sperm count, motility (PM and IM) and morphology, Spearman's correlation analysis was performed. The correlation coefficients, with adjusted p-values, were consistent in screening as well as validation phases. Most importantly, a negative correlation between miRNAs and sperm count, PM, and morphology but positive correlation with IM sperm was observed, as illustrated in Supplemental Fig. 5. Among numerous miRNAs assessed, three were chosen particularly because of their highly significant and strong correlations with sperm count, PM, IM, and M (adjusted p < 0.001) (Fig. 4F). The three miRNAs, hsa-miR-15b-5p, hsa-miR-19a-5p, and hsa-miR-20a-5p, exhibited the same correlation patterns throughout the study.

Interestingly, these three miRNAs were consistently detected in various types of sperm (i.e., OA, A, OAT, and AT) (Fig. 4). Specifically, hsa-miR-15b-5p was significantly negatively correlated with sperm count (r = −0.668), motility (r = −0.761), and morphology (r = −0.444), and was also significantly positively correlated in IM sperm (r = 0.636). Likewise, hsa-miR-19a-5p was significantly negatively correlated with sperm count (r = −0.704), PM (r = −0.794), and morphology (r = −0.434), and was also positively correlated with IM (r = 0.578). Similarly, hsa-miR-20a-5p had inverse correlations with sperm count (r = −0.675), PM (r = −0.766), and morphology (r = −0.425) but a positive correlation with IM (r = 0.645); all the correlations were statistically significant (adjusted p < 0.001).

#### 3.6.5 Relationship between miRNA expression levels and FSH, LH, embryo quality, and β-hCG/live birth rates

Spearman's correlation was used to examine the correlation between miRNA levels of expression and parameters such as FSH, LH, β-hCG, embryo grades, and IVF results (live birth). A weak positive correlation for LH and FSH was observed in 10 out of the 16 miRNAs screened by RT-qPCR, as demonstrated in Supplemental Fig. 6. Amongst them, the miRNAs hsa-miR-15b-5p (r = 0.466), hsa-miR-20a-5p (r = 0.453), hsa-miR-16-5p (r = 0.414), and hsa-miR-449b-5p (r = 0.453) presented the highest correlation coefficients. Additionally, hsa-miR-19a-5p presented a weaker but statistically significant correlation (r = 0.387, adjusted p < 0.001).

In the same way, the same trend was observed in LH hormone levels, and positive weak correlations were detected for hsa-miR-15b-5p (r = 0.432), hsa-miR-19a-5p (r = 0.335), and hsa-miR-20a-5p (r = 0.425), as presented in Supplemental Fig. 6 miRNA expression analysis according to β-hCG levels, embryo grading, and IVF outcomes showed significant variations between clinical situations. All of the miRNAs followed the same pattern, having high expression levels in the negative β-hCG group as compared to the positive β-hCG group.

In particular, hsa-miR-15b-5p, hsa-miR-19a-5p, hsa-miR-20a-5p, and hsa-miR-449b-5p had significantly higher expression levels in the negative β-hCG group (adjusted p values of 0.0053, 0.0486, 0.0076, and 0.0442, respectively) with log2 fold changes (log2FC) of 1.21, 1.10, 1.21, and 1.25, respectively, as indicated in Fig. 4A. In embryo grade analysis, the samples were divided into four grades: G1, G2, G3, and G4 (Fig. 4B). The comparison of the expression levels of these miRNAs revealed significant differences, with hsa-miR-15b-5p and hsa-miR-20a-5p having lower expression in G1 compared to G2 (adjusted p = 0.0151, log2FC = 1.21 and adjusted p = 0.0158, log2FC = 1.21), suggesting their role in the development of embryos and embryo quality assessment. For the comparison of successful and unsuccessful IVF outcomes (live birth versus failed IVF cycles or negative pregnancy outcomes), a total of 85 samples were used. Of these samples, 14 had no reported outcome, 30 were successful outcomes, and 41 had negative β-hCG levels and therefore were unsuccessful IVF outcomes. Three specific miRNAs, hsa-miR-15b-5p (adjusted p = 0.00292, log2FC = 1.22), hsa-miR-19a-5p (adjusted p = 0.0411, log2FC = 1.10), and hsa-miR-20a-5p (adjusted p = 0.00583, log2FC = 1.22), had significantly higher expression levels when there were failed IVF outcomes versus successful live births (Fig. 4C). This was a finding irrespective of whether the pregnancy resulted in a single birth or the birth of twins. The study indicates that elevated levels of these miRNAs can be linked to less successful outcomes in in vitro fertilization.

#### 3.6.6 Diagnostic significance of selected miRNAs

To assess the diagnostic potential of miRNAs that were significantly correlated with sperm parameters and positive IVF outcomes (live birth), we estimated their AUC and evaluated their overall diagnostic performance in distinguishing between positive and negative samples. As shown in Fig. 4D, the predictive power of three miRNAs **hsa-miR-15b-5p, hsa-miR-19a-5p,** and **hsa-miR-20a-5p** was assessed using logistic regression models, with AUC values determined by ROC curve analysis. The AUC values were **0.76 for hsa-miR-15b-5p** (95 % CI: 0.64–0.87, p = 9.11 × 10^−5^), **0.71 for hsa-miR-19a-5p** (95 % CI: 0.59–0.83, p = 0.0013), and **0.74 for hsa-miR-20a-5p** (95 % CI: 0.63–0.86, p = 0.0002). A combined logistic regression model incorporating all three miRNAs yielded an **overall AUC of 0.75** (95 % CI: 0.63–0.86, p = 0.0001). These results highlight the potential of these miRNAs, individually or in combination, as biomarkers for distinguishing successful from unsuccessful IVF outcomes.

## 4 Discussion

In this study, we conducted comprehensive profiling of small RNAs in sperm samples from 13 male partners of couples undergoing fertility treatment. Each participant provided three samples comprising 1500 individually selected sperm, categorized based on motility and morphology. The study was later expanded to include 85 additional participants, from whom bulk sperm samples were collected without single-cell selection, enabling validation of small RNA sequencing data via RT-qPCR. Our data support previous findings that abnormalities in sperm morphology and motility can negatively impact embryo development and viability, ultimately compromising assisted reproduction outcomes [23,24]. This emphasizes the need for a more thorough evaluation of male contributions to IVF success and the identification of molecular biomarkers beyond routine semen analysis.

In agreement with earlier research, our analysis across both cohorts revealed that higher sperm count, progressive motility, and normal morphology were strongly associated with improved embryo quality and higher pregnancy rates [25,26]. Furthermore, favorable pregnancy outcomes such as clinical pregnancy or live birth were positively correlated with these parameters, along with lower FSH and LH levels [[25], [26], [27]]. Conversely, unsuccessful outcomes including implantation failure or miscarriage were linked to reduced motility and elevated hormone levels. While these factors are part of standard fertility evaluations, they may fail to detect idiopathic infertility or cases where sperm parameters appear normal yet still compromise ART outcomes. This gap highlights the need for novel molecular biomarkers to enhance diagnostic precision.

This study demonstrates that small non-coding RNAs particularly miRNAs and piRNAs are strongly associated with sperm quality and male fertility. We identified distinct expression profiles between sperm with normal versus poor motility and morphology, supporting the hypothesis that small RNAs carried by sperm influence reproductive potential [6,28]. Mature sperm, though transcriptionally inert, contain a rich repertoire of small RNAs, suggesting these molecules play functional roles in sperm maturation, fertilization, and early embryogenesis [6,[28], [29], [30]]. Our results show that dysregulated expression of specific miRNAs and piRNAs correlates with poor semen quality and impaired fertilization. Sperm with higher motility and normal morphology produced embryos of superior quality, while poor motility and abnormal forms were linked to lower embryo quality and reduced blastocyst formation. Even in ICSI cases, where fertilization is assisted, embryos derived from morphologically abnormal sperm showed increased aneuploidy and compromised development [31,32]. These findings emphasize the critical contribution of sperm integrity to embryogenesis and implantation success.

Among the miRNAs analyzed, miR-15b-5p, miR-19a-5p, and miR-20a-5p were significantly overexpressed in sperm samples with poor motility and morphology. This overexpression correlated with lower embryo quality scores and unsuccessful pregnancy outcomes, suggesting that these miRNAs may serve as negative fertility indicators. miR-19a-5p and miR-20a-5p belong to the miR-17–92 cluster, a polycistronic group known to regulate germ cell proliferation and apoptosis [33]. Dysregulation of this cluster has been linked to infertility; for example, miR-17–92 deletion in mice results in oligozoospermia and reduced motility [34]. Their elevated levels in our study may reflect compensatory responses to spermatogenic stress or incomplete sperm maturation. Likewise, Tomic et al. reported miR-15b downregulation in teratozoospermic men [35], suggesting a role in healthy spermatogenesis. In contrast, our study found miR-15b-5p overexpressed in poor-quality sperm, which may reflect cohort differences or distinct infertility etiologies. Nonetheless, both studies underscore miR-15b′s importance in sperm development.

Our results align with prior research connecting sperm miRNA profiles with embryo development. Abu-Halima et al. (2020) reported that hsa-miR-19b-3p was lower in sperm from couples who achieved pregnancy [14], supporting our findings on its paralog, miR-19a. Additional studies have identified miR-191-5p and others as markers of high-grade embryos [36], reinforcing the role of small RNAs in assessing paternal reproductive fitness. Consequently, elevated expression of specific miRNAs such as miR-15b, miR-19a/b, and miR-20a may interfere with post-fertilization gene expression by inappropriately silencing key developmental regulators in the zygote.

In parallel, we analyzed piRNA expression and observed higher levels in poor-quality sperm, especially in Group C (completely immotile, morphologically defective). piRNAs are small non-coding RNAs best known for suppressing transposable elements in the germline but also participate in post-transcriptional mRNA regulation during spermatogenesis [6,9,37]. Their dysregulation has been increasingly linked to sperm dysfunction [38]. piRNAs accounted for 5.96 % of total aligned reads in Group C, compared to 3.72 % in Group A (normal morphology/motility), suggesting a possible overactivation of the piRNA pathway in response to genomic instability. While the function of piRNAs post-fertilization remains under investigation, their dysregulation may influence zygotic genome activation and transcript clearance. miRNA expression, by contrast, was reduced across quality groups: 0.83 % in Group A, 0.76 % in Group B, and 0.66 % in Group C. This decline may reflect disrupted gene regulation and a shift to a dysfunctional sperm state.

To assess clinical relevance, ROC curve analyses shown in Fig. 4 demonstrated that miR-15b-5p, miR-19a-5p, and miR-20a-5p possessed strong predictive power in distinguishing successful from failed IVF cycles. Although preliminary due to limited sample size, these findings align with those of Chen et al. (2023), who demonstrated that miRNA profiling combined with clinical parameters improved pregnancy prediction (AUC ∼0.85 vs. ∼0.75) [39]. A meta-analysis by Huang et al. (2023) similarly reported a pooled AUC of 0.81 for small RNA biomarkers predicting embryo implantation [40], with miR-20a being among the most consistent markers. Collectively, these studies suggest that sperm-derived RNAs—including both miRNAs and piRNAs—have strong potential as prognostic biomarkers. Integrating sperm RNA profiling with embryonic and endometrial information could substantially improve the precision of embryo selection strategies.

Further differential expression analysis identified additional miRNAs of interest. Decreased miR-223 in embryo culture media has been associated with poor IVF/ICSI outcomes [41]. miR-424, linked to elevated sperm DNA fragmentation, correlates with its target gene SPAG7 essential for sperm function [42,43]. Altered miR-424 and SPAG7 levels have been observed in oligoasthenozoospermia [43]. Additional candidates include miR-199a-5p and miR-99a-5p, which predict pregnancy failure post-Day 5 development [44]. miR-199a-5p has also been implicated in flagellar defects during spermiogenesis [45]. The miR-449 family is vital for germ cell function and cilium assembly; deletion in mice leads to oligoasthenoteratozoospermia [46]. Upregulation of miR-16, miR-19a, and miR-15b has been reported in various infertility conditions [[47], [48], [49]]. miR-106b-5p promotes spermatogonial stem cell proliferation [50]. miR-146a-5p distinguishes obstructive from secretory azoospermia [51]. miR-16 is also abundant in sperm and embryo culture media, playing a key role in early development and gene regulation [[47], [48], [49],^52^].

From a clinical standpoint, developing high-throughput assays or qPCR panels for deleterious miRNAs and piRNAs could enhance diagnostic workflows. A composite “small RNA risk score” might help identify individuals with reduced IVF success likelihood, informing treatment choices like ICSI, preimplantation genetic testing, or antioxidant therapy. However, standardization across RNA extraction methods, sequencing platforms, and patient populations is essential. Rigorous validation via multicenter trials is necessary to confirm diagnostic utility. Functional studies such as miRNA knockdown or gene editing are also needed to establish causality.

While paternal age is recognized as a contributor to reduced fertility and adverse offspring outcomes [53], prior studies have shown miRNA expression to be influenced by age and sex [54]. In contrast, no significant association was observed between paternal age and miRNA levels, suggesting that changes in expression were more closely linked to sperm count, motility, morphology, and hormone levels (FSH, LH, β-hCG), as well as embryo quality and ART outcomes.

Lastly, miR-15b-5p, miR-19a-5p, and miR-20a-5p previously implicated in spermatogenesis and male fertility showed strong associations with reduced sperm quality in our study. Tomic et al. reported reduced miR-15b-5p in teratozoospermic sperm [35], while miR-19a-5p and miR-20a-5p, part of the miR-17 family, are known regulators of early development and stem cell differentiation [55]. miR-20a-5p has also been detected in spent blastocyst media and may act as a non-invasive marker of embryo potential [56]. Our findings demonstrate that miR-15b and miR-19a levels are inversely associated with sperm count, motility, and morphology, supporting their potential as biomarkers of male fertility. Additionally, miRNAs originating from sperm, seminal plasma, and embryo culture media appear to be valuable indicators of sperm quality and may serve as predictive markers of reproductive outcomes.

In support of these conclusions, miR-19a and miR-15b present in sperm and seminal plasma have been proposed as markers of impaired spermatogenesis [48,57]. miR-19b-3p, which shares targets with miR-19a, was downregulated in sperm associated with positive pregnancy outcomes [14]. miR-20a-5p has also shown utility in embryo grading, offering a higher AUC when comparing Grade 2 and Grade 3 embryos [14], supporting its role as a functional biomarker for sperm quality and embryonic developmental potential.

This study has several limitations that warrant attention. A significant concern is the limited size of the study population, which consisted of a modest number of participants. Specifically, 13 men in the single-sperm discovery cohort (Groups A–C) and 85 individuals in the independent validation cohort, all of whom were recruited from a singular infertility center. These constraints could potentially diminish statistical power and the ability to generalize findings, necessitating larger multicenter investigations to validate the results obtained. Furthermore, maternal factors were not included as selection or adjustment variables in the study design. Although the age range of female partners (20–30 years) may reduce some variability in outcomes, other factors such as body mass index (BMI), ovarian reserve, stimulation protocols, and uterine conditions could significantly affect embryo quality and pregnancy results; thus, the absence of such data introduces a risk of residual confounding. In addition, while our three-miRNA panel (hsa-miR-15b-5p, hsa-miR-19a-5p, hsa-miR-20a-5p) demonstrated potential in augmenting conventional evaluations of sperm quality and in IVF prognosis, further validation within larger and more heterogeneous cohorts is essential prior to clinical application. Lastly, we refrained from conducting in-silico target prediction or pathway enrichment analyses, instead prioritizing robust clinical associations with well-characterized miRNAs. Although this approach mitigates speculative interpretations, it also limits the mechanistic depth of our findings; thus, future research combining functional validation with integrated bioinformatics methodologies is essential.

## 5 Conclusion

This study provides novel insights into the molecular landscape of sperm and its relationship with semen quality, embryo development, and reproductive outcomes. We demonstrate that conventional sperm parameters particularly motility and morphology are closely associated with embryo developmental competence and pregnancy success. Importantly, we extend this association to the molecular level by identifying specific small RNAs especially miR-15b-5p, miR-19a-5p, and miR-20a-5p that consistently correlate with sperm quality, hormonal profiles, embryo grading, and IVF outcomes.

Our small RNA sequencing revealed a diverse repertoire of RNA classes in sperm, with long non-coding RNAs being the most abundant. Among these, miRNAs and piRNAs demonstrated the strongest associations with key reproductive parameters. These findings are consistent with previous studies and suggest that aberrant expression of specific miRNAs may reflect underlying defects in spermatogenesis or sperm maturation, contributing to subfertility. While the mechanistic roles of these RNAs remain to be fully elucidated, our results indicate their potential as non-invasive biomarkers for evaluating male reproductive potential.

Taken together, our study highlights the value of integrating molecular markers such as small RNAs into existing assessments of male fertility and IVF prognosis. Although further validation in larger and more diverse cohorts is needed, these findings offer promising avenues for improving the diagnosis and clinical management of male-factor infertility, as well as enhancing the precision of ART.

## CRediT authorship contribution statement

**Mohammad A. Al Smadi:** Visualization, Validation, Methodology, Investigation, Data curation. **Haidar Haidar:** Visualization, Validation, Methodology, Investigation. **Albert Salas-Huetos:** Writing – review & editing, Visualization, Supervision. **Ulrike Fischer:** Writing – review & editing, Supervision. **Hashim Abdul-Khaliq:** Supervision, Resources, Project administration. **Eckart Meese:** Writing – review & editing, Supervision, Resources, Project administration. **Masood Abu-Halima:** Writing – review & editing, Writing – original draft, Visualization, Validation, Supervision, Software, Resources, Project administration, Methodology, Investigation, Funding acquisition, Formal analysis, Data curation, Conceptualization.

## Ethics approval and consent to participate

The study was approved by the Institutional Review Board of the Saarland Medical Association/Germany (Ha 195/11/updated June 2021 and NF. 52/25 Saarland University) and was conducted according to ethical guidelines and the Declaration of Helsinki. Written informed consent was obtained from all participants.

## Data sharing statement

The datasets generated and/or analyzed during this study are available from the corresponding author upon reasonable request. Furthermore, all raw data from the Small RNA sequencing experiments have been deposited in the NCBI Sequence Read Archive (SRA) in FASTQ format under the accession ID: SUB15458828 and BioProject ID: PRJNA1291729.

## Funding

This study was funded by Hedwig-Stalter foundation (2016)

## Declaration of competing interest

The authors declare that they have no known competing financial interests or personal relationships that could have appeared to influence the work reported in this paper.

## Appendix A Supplementary data

Supplemental Figure 1| Correlation of miRNA Expression Levels with Sperm Parameters. Scatter plots show the correlation between miRNA expression levels and sperm parameters. Each plot provides the Spearman correlation coefficient (r) and p-value for the respective miRNA and parameter. Statistical significance was determined using adjusted p-value threshold of <0.05. Supplemental Figure 2| Correlation of piRNA Expression Levels with Sperm Parameters. Scatter plots show the correlation between piRNA expression levels and sperm parameters. Each plot provides the Spearman correlation coefficient (r) and p-value for the respective piRNA and parameter. Statistical significance was determined using adjusted p-value threshold of <0.05. Supplemental Fig. 3| Correlation of miRNA Expression Levels with Age. Scatter plots show the correlation between miRNA expression levels and age (in years). The y-axis represents miRNA expression levels, while the x-axis denotes age in years. Each plot provides the Spearman correlation coefficient (r) and p-value for the respective miRNA and parameter. Statistical significance was determined using adjusted p-value threshold of <0.05. Supplemental Figure 4| Differential miRNA Expression Across Sperm Classification Groups. The miRNA expression levels (ΔCt) are compared across sperm classification groups: Oligoasthenospermia (OA), Asthenozoospermia (A), Oligoasthenoteratozoospermia (OAT), and Asthenoteratozoospermia (AT). Each plot displays miRNA expression levels (ΔCt) for each classification group. Statistical significance (p-values) is shown for each miRNA. Lower ΔCt indicates a higher abundance level. Supplemental Figure 5| Correlation of miRNA Expression with Sperm Parameters. Scatter plots show the correlation between miRNA expression levels and sperm parameters. The Spearman correlation coefficient (r) and p-values are provided for each plot. Statistical significance was determined using adjusted p-value threshold of <0.05. Supplemental Figure 6| Correlation of miRNA Expression Levels with Hormonal Parameters (FSH and LH). Scatter plots show the correlation between miRNA expression levels and hormonal parameters, including follicle-stimulating hormone (FSH) and luteinizing hormone (LH). The p-values and Spearman correlation coefficient (r) are provided for each plot. Statistical significance was determined using adjusted p-value threshold of <0.05.Multimedia component 1.
