# Supplementary material for: Small RNA sequencing in individually selected sperm: Biomarkers for male subfertility and predictors of pregnancy success
Source: Noncoding RNA Res. 2025 Oct 10;16:126–43. doi: 10.1016/j.ncrna.2025.09.008 (PMC12648719; doi:10.1016/j.ncrna.2025.09.008)
Supplement: Supplementary file 1 — Supplemental Figure 1| Correlation of miRNA Expression Levels with Sperm Parameters. Scatter plots show the correlation between miRNA expression levels and sperm parameters. Each plot provides the Spearman correlation coefficient (r) and p-value for the respective miRNA and parameter. Statistical significance was determined using adjusted p-value threshold of <0.05. Supplemental Figure 2| Correlation of piRNA Expression Levels with Sperm Parameters. Scatter plots show the correlation between piRNA expression levels and sperm parameters. Each plot provides the Spearman correlation coefficient (r) and p-value for the respective piRNA and parameter. Statistical significance was determined using adjusted p-value threshold of <0.05. Supplemental Fig. 3| Correlation of miRNA Expression Levels with Age. Scatter plots show the correlation between miRNA expression levels and age (in years). The y-axis represents miRNA expression levels, while the x-axis denotes age in years. Each plot provides the Spearman correlation coefficient (r) and p-value for the respective miRNA and parameter. Statistical significance was determined using adjusted p-value threshold of <0.05. Supplemental Figure 4| Differential miRNA Expression Across Sperm Classification Groups. The miRNA expression levels (ΔCt) are compared across sperm classification groups: Oligoasthenospermia (OA), Asthenozoospermia (A), Oligoasthenoteratozoospermia (OAT), and Asthenoteratozoospermia (AT). Each plot displays miRNA expression levels (ΔCt) for each classification group. Statistical significance (p-values) is shown for each miRNA. Lower ΔCt indicates a higher abundance level. Supplemental Figure 5| Correlation of miRNA Expression with Sperm Parameters. Scatter plots show the correlation between miRNA expression levels and sperm parameters. The Spearman correlation coefficient (r) and p-values are provided for each plot. Statistical significance was determined using adjusted p-value threshold of <0.05. Supplemental F [file mmc1.pptx]

## Slide 1
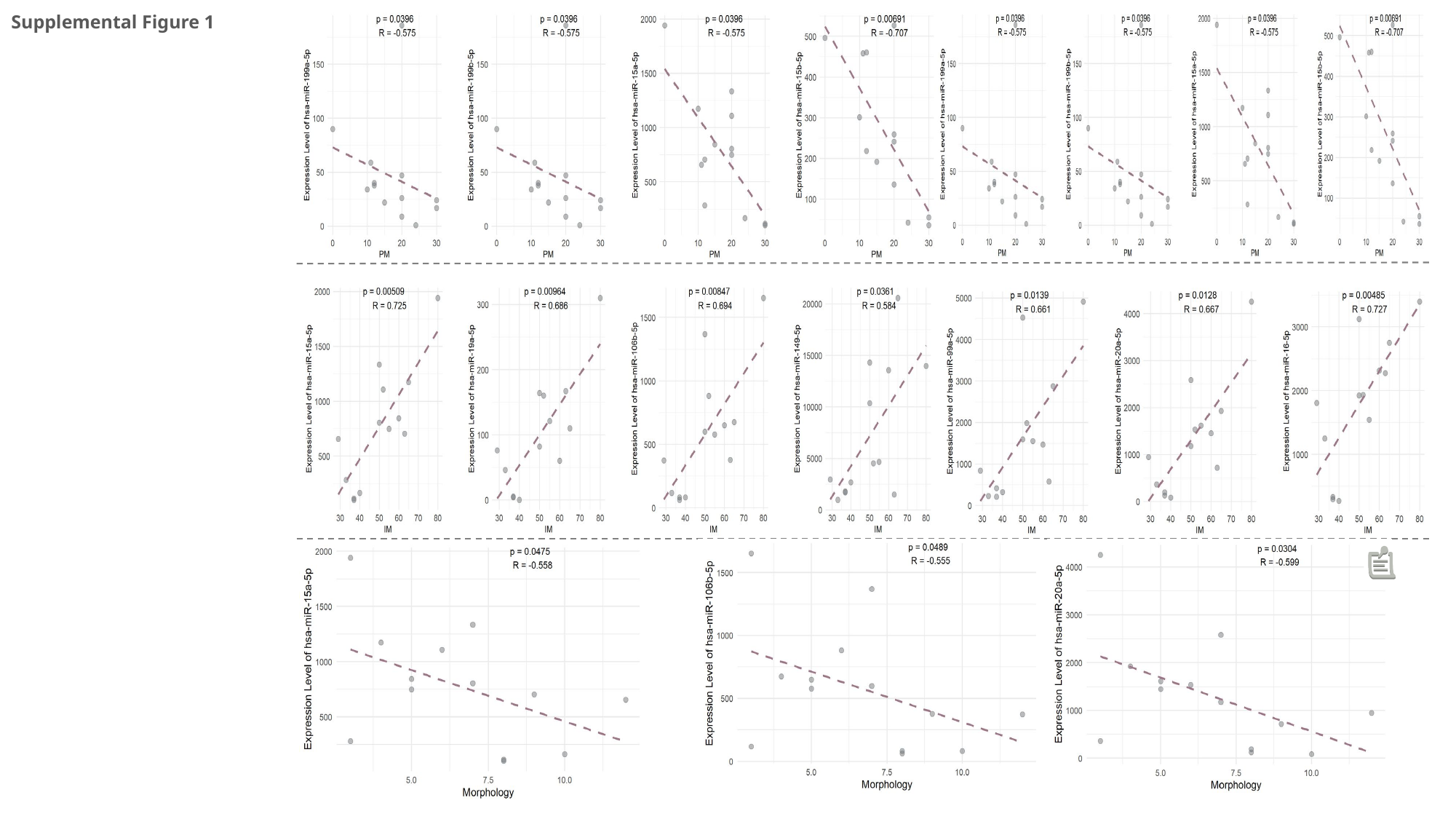

Supplemental Figure 1

## Slide 2
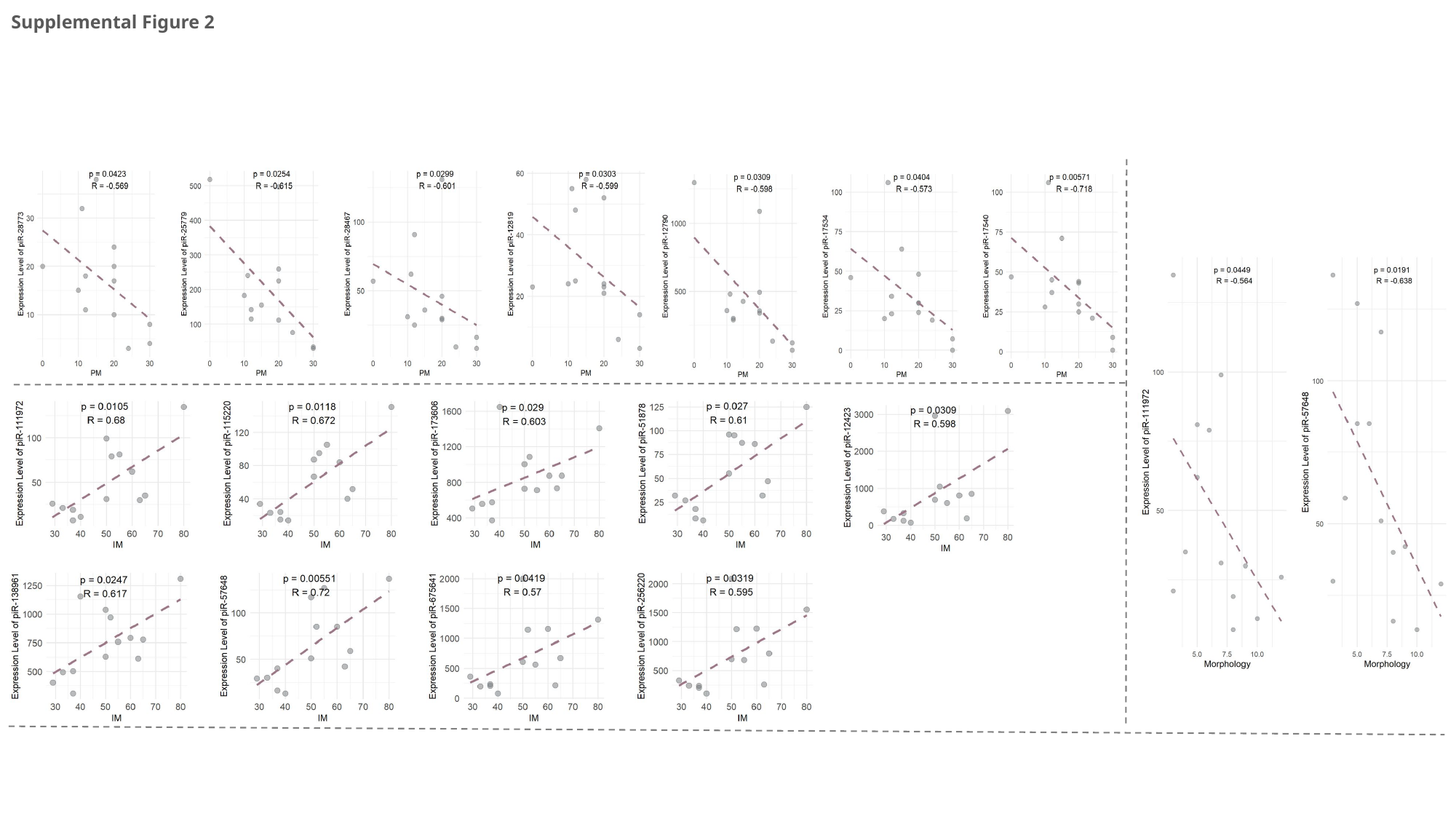

Supplemental Figure 2

## Slide 3
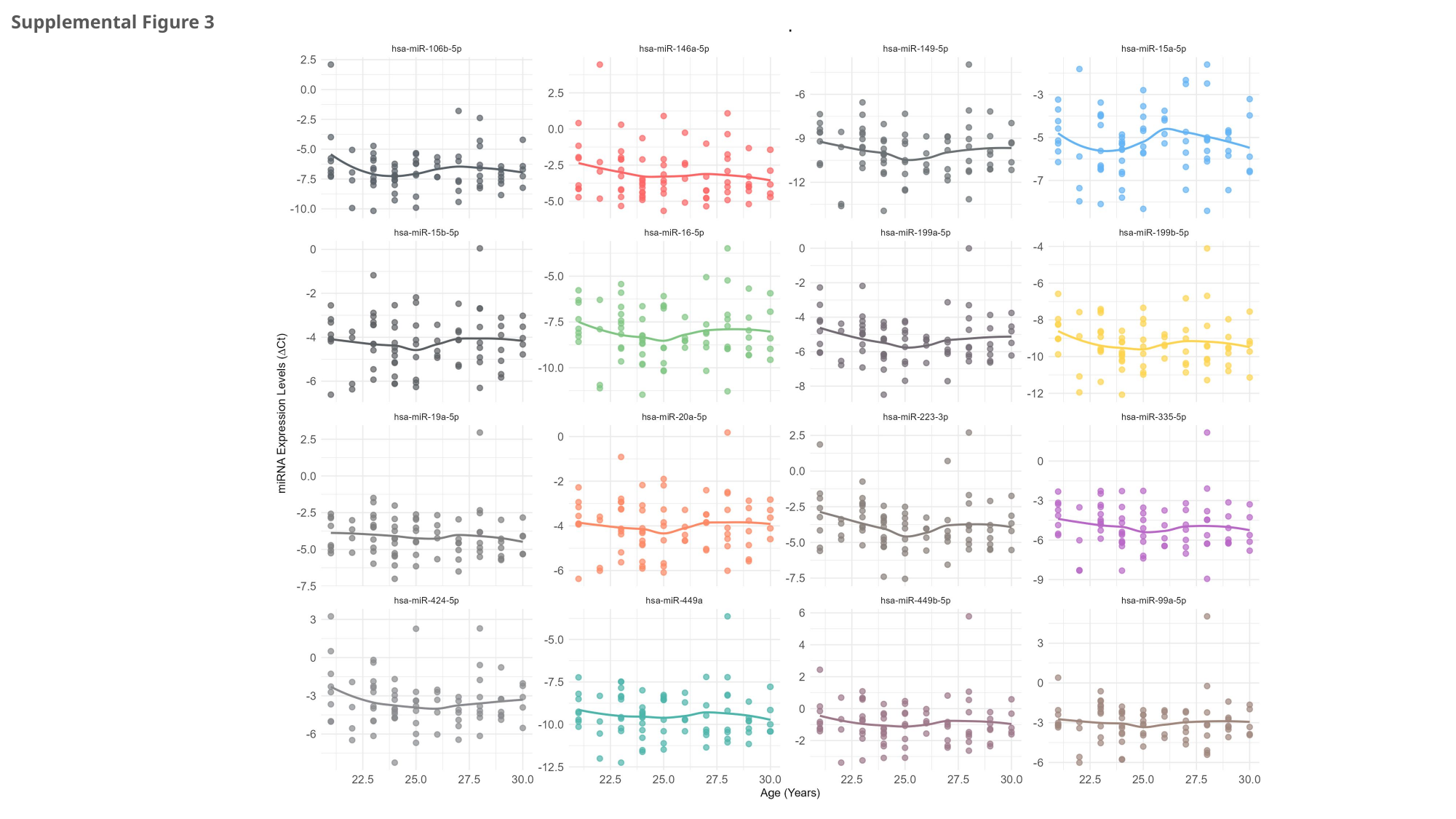

Supplemental Figure 3

## Slide 4
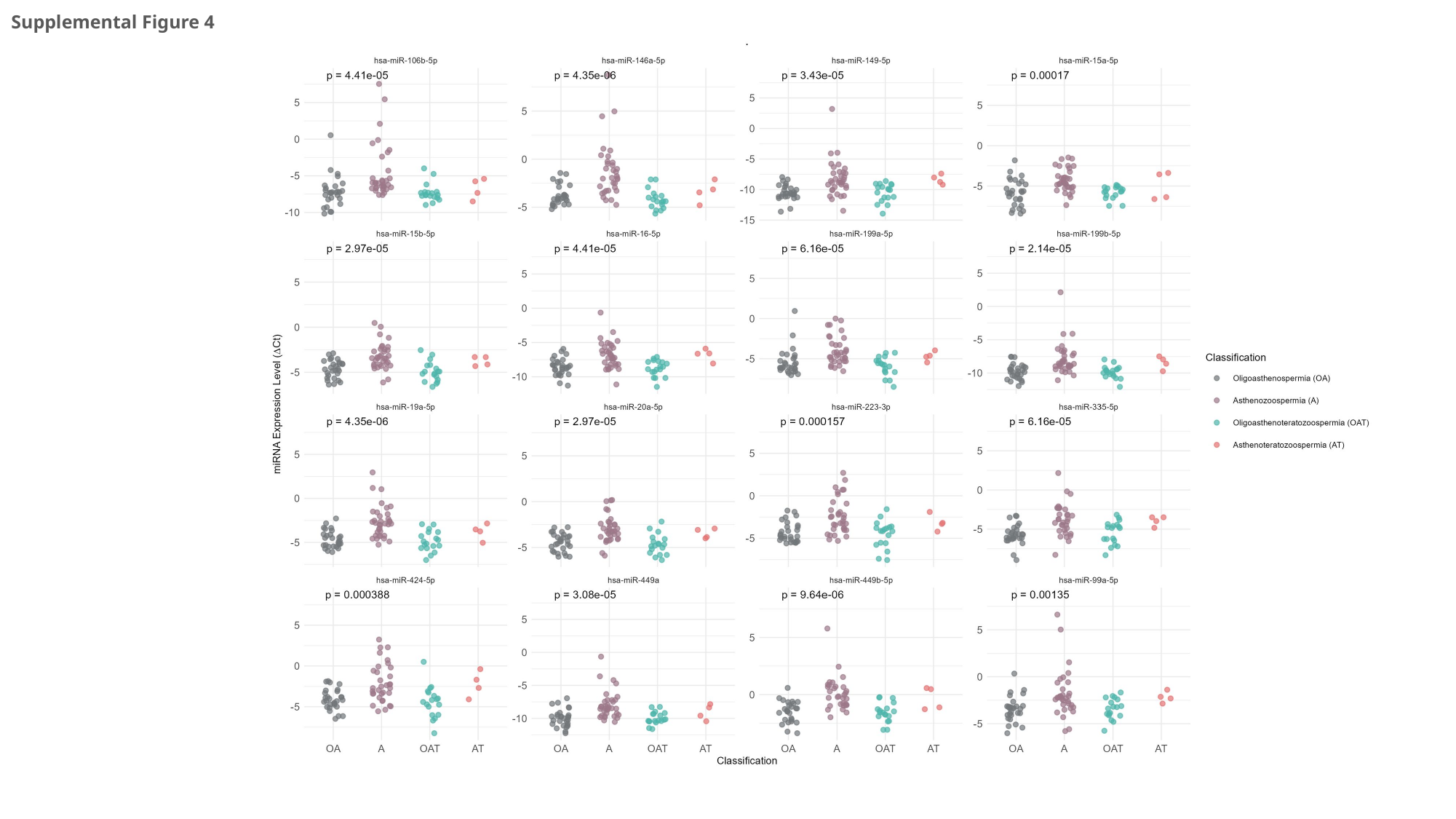

Supplemental Figure 4

## Slide 5
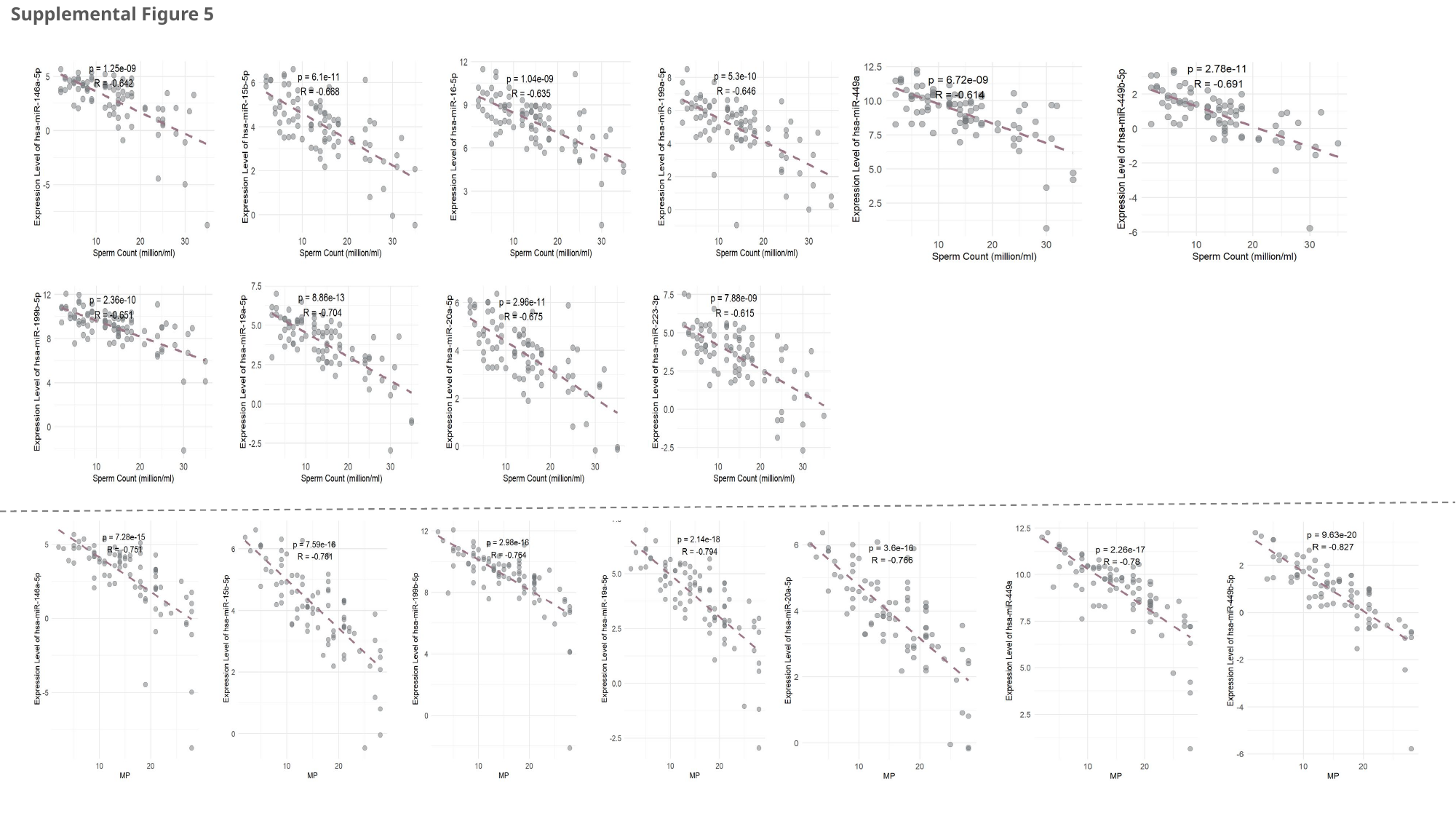

Supplemental Figure 5

## Slide 6
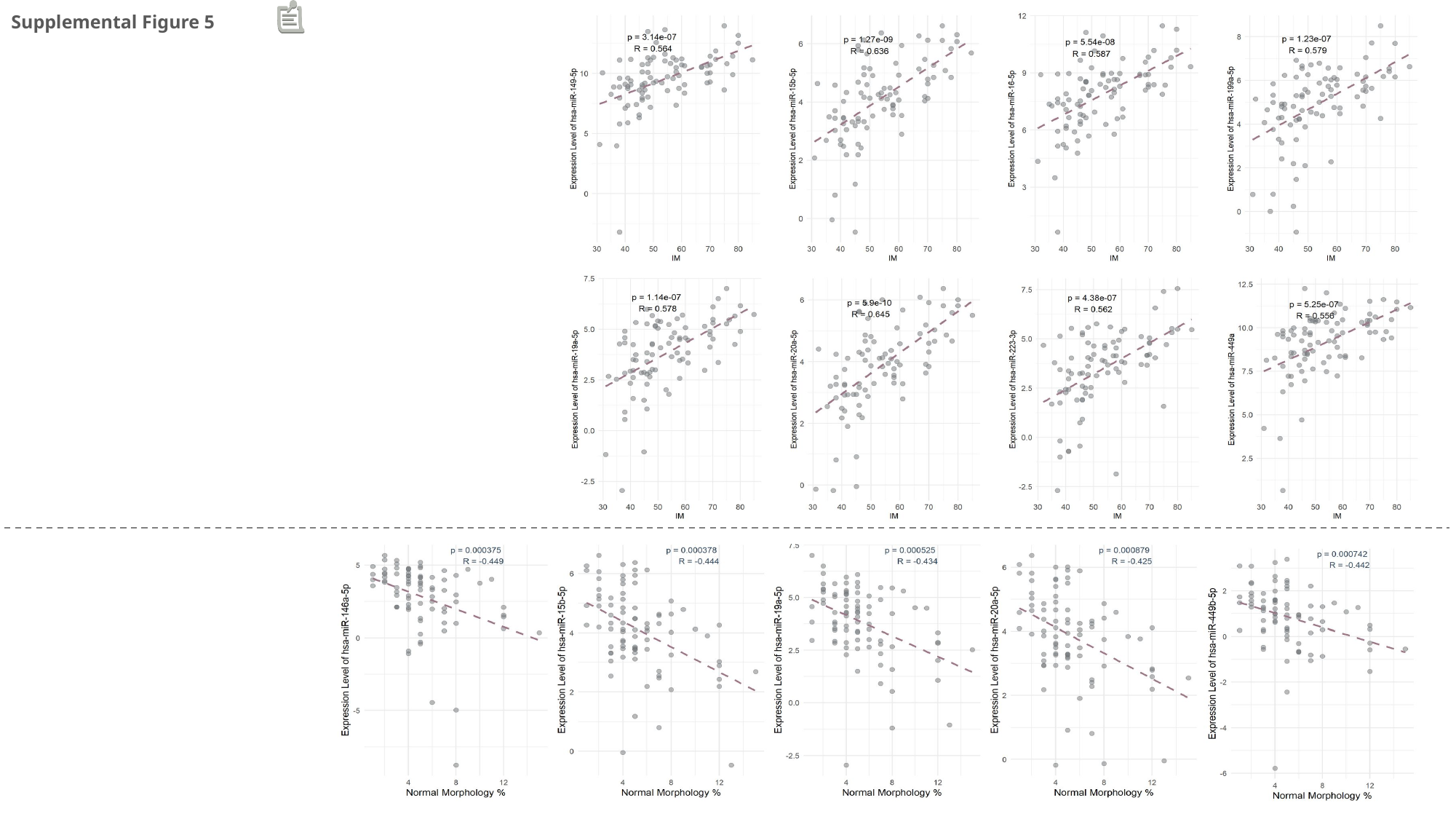

Supplemental Figure 5

## Slide 7
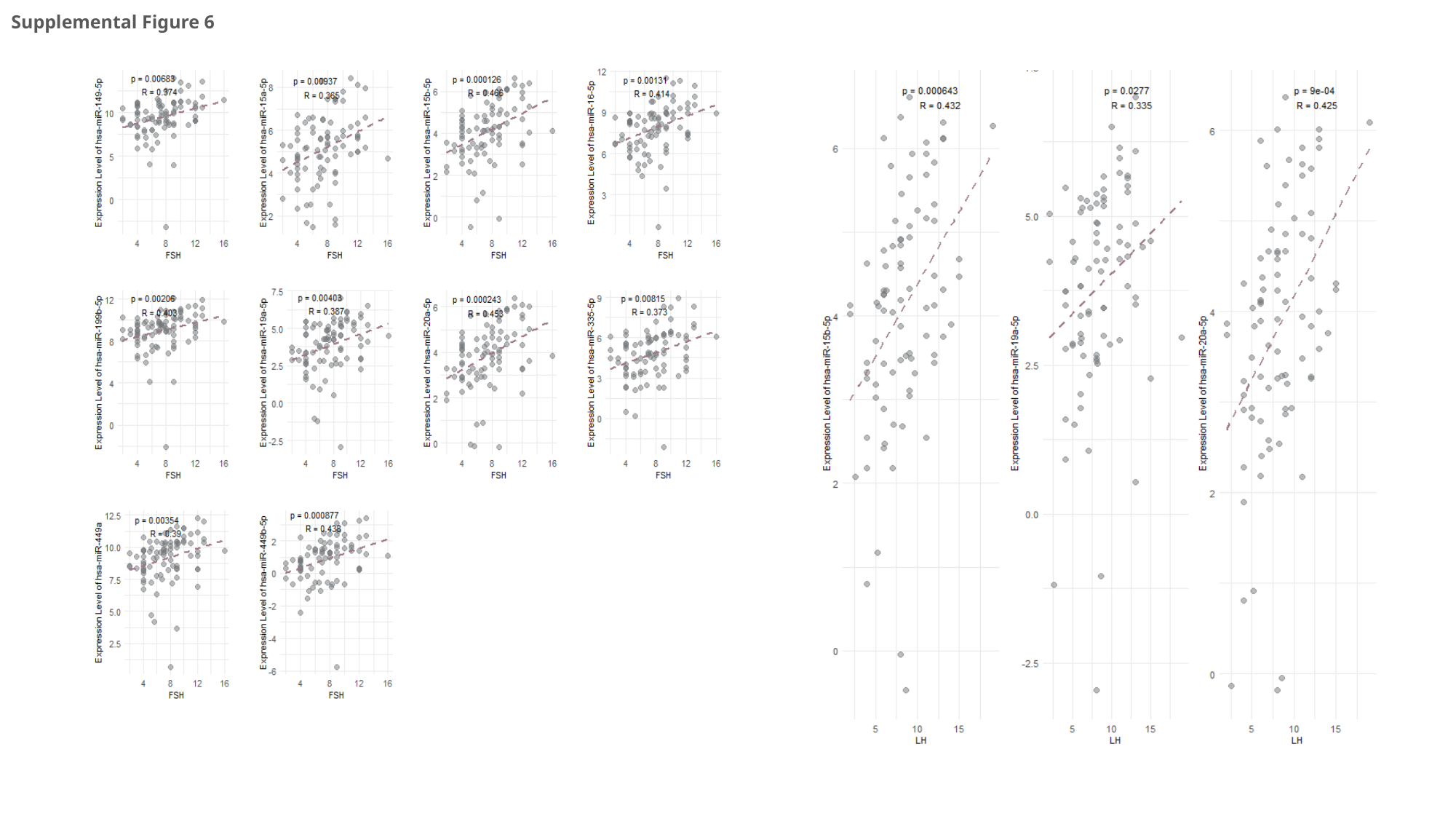

Supplemental Figure 6
