# Supplementary material for: Small RNA sequencing in individually selected sperm: Biomarkers for male subfertility and predictors of pregnancy success
Source: Noncoding RNA Res. 2025 Oct 10;16:126–43. doi: 10.1016/j.ncrna.2025.09.008 (PMC12648719; doi:10.1016/j.ncrna.2025.09.008)
Supplement: Multimedia component 2 [file mmc2.docx]

**Supplementary Tables**

**Supplementary Table 1|** Clinical Information for Participants Included in Cohort 1 for Small RNA-Sequencing Analysis.

| Male Partner Sperm Parameters | | | | | | | IVF Outcome Parameters | Female Partner Hormonal Parameters | | | |
| --- | --- | --- | --- | --- | --- | --- | --- | --- | --- | --- | --- |
| Male ID | **Age**  **(Year)** | **Sperm Count (million/ml)** | **Progressive Motility (Type A+B) %** | **Non-Progressive Motility (Type C) %** | **Immotile Sperm (Type D) %** | **Normal Morphology (%)** | **Embryo Quality Grade** | **β-hCG** | **Live Birth Outcome** | **FSH**  **(mIU/ml)** | **LH**  **(mIU/ml)** |
| 1 | 22 | 13 | 12 | 55 | 33 | 3 | G2 | Negative | Negative | 6.4 | 9.3 |
| 2 | 29 | 55 | 30 | 33 | 37 | 8 | G1 | Positive | Boy | 5.6 | 7.1 |
| 3 | 30 | 14 | 10 | 25 | 65 | 4 | G2 | Positive | Boy/Girl | 2.8 | 6.8 |
| 4 | 20 | 55 | 20 | 25 | 55 | 5 | G1 | Positive | Abortion | 2.2 | 3.5 |
| 5 | 26 | 40 | 20 | 30 | 50 | 7 | No Oocyte Retrieval | Negative | Negative | 7.5 | 10.2 |
| 6 | 25 | 18 | 0 | 20 | 80 | 3 | G3 | Negative | Negative | 8.6 | 11.4 |
| 7 | 30 | 40 | 11 | 60 | 29 | 12 | G1 | Positive | Girl | 3.3 | 8.7 |
| 8 | 24 | 42 | 20 | 28 | 52 | 6 | G2 | Negative | Negative | 8.9 | 8.6 |
| 9 | 28 | 20 | 15 | 25 | 60 | 5 | No Cleavage | Negative | Negative | 5.47 | 6.57 |
| 10 | 23 | 45 | 12 | 25 | 63 | 9 | G1 | Positive | Girl | 4.6 | 5.8 |
| 11 | 21 | 65 | 20 | 30 | 50 | 7 | G1/G2 | Negative | Negative | 7.8 | 11.6 |
| 12 | 27 | 50 | 24 | 36 | 40 | 10 | G1/G2 | Negative | Negative | 9.7 | 4.3 |
| 13 | 23 | 52 | 30 | 33 | 37 | 8 | G1 | Positive | Negative | 10.2 | 9.8 |
| Mean (± SD) | **25.23 (± 3.39)** | **39.15**  **(±17.39)** | **17.23**  **(± 8.42)** | **32.69**  **(±11.86)** | **50.08**  **(±14.69)** | **6.69**  **(±2.72)** | **-** | **-** | **-** | **6.39 (±2.65)** | **7.97 (±2.55)** |

**Abbreviation:** Beta Human Chorionic Gonadotropin (β-hCG), Follicle-Stimulating Hormone (FSH), Luteinizing Hormone (LH), Grade 1 (G1), Grade 2 (G2)

**Supplementary Table 2|** Clinical Information for Participants Included in Cohort 2 for RT-qPCR Analysis.

| Male Partner Sperm Parameters | | | | | | | | | IVF Outcome Parameters | Female Partner Hormonal Parameters | | | |
| --- | --- | --- | --- | --- | --- | --- | --- | --- | --- | --- | --- | --- | --- |
| Male ID | **Age**  **(Year)** | **Sperm Count (million/mL)** | **Progressive Motility (Type A+B) %** | **Non-Progressive Motility (Type C) %** | **Immotile Sperm (Type D) %** | **Normal Morphology %** | **Classification** | **Embryo**  **Quality Grade** | | **β-hCG** | **FSH (mIU/ml)** | **LH**  **(mIU/ml)** | **Live Birth Outcome** |
| 1 | 25 | 7 | 9 | 30 | 61 | 5 | Oligoasthenospermia | 2G1 | | Negative | 7.2 | 9.3 | NA |
| 2 | 23 | 6 | 11 | 40 | 49 | 3 | Oligoasthenoteratozoospermia | 2G2 | | Negative | 7.3 | 5.8 | NA |
| 3 | 30 | 6 | 14 | 35 | 51 | 5 | Oligoasthenospermia | 1G1/1G2 | | Negative | 5.5 | 8.6 | NA |
| 4 | 27 | 25 | 28 | 31 | 41 | 7 | Asthenozoospermia | 3G1 | | Negative | 8.3 | 7.5 | NA |
| 5 | 26 | 26 | 18 | 34 | 48 | 6 | Asthenozoospermia | 2G2 | | Negative | 7.4 | 4.9 | NA |
| 6 | 23 | 7 | 9 | 22 | 69 | 2 | Oligoasthenoteratozoospermia | 2G2 | | Negative | 7.3 | 8.1 | NA |
| 7 | 23 | 15 | 21 | 32 | 47 | 6 | Asthenozoospermia | 2G2 | | Negative | 8.4 | 6.4 | NA |
| 8 | 26 | 14 | 18 | 34 | 48 | 3 | Oligoasthenoteratozoospermia | 2G1 | | Negative | 9.8 | 8.6 | NA |
| 9 | 23 | 12 | 18 | 40 | 42 | 3 | Oligoasthenoteratozoospermia | 2G1 | | Negative | 7.0 | 8.5 | NA |
| 10 | 25 | 16 | 21 | 38 | 41 | 4 | Asthenozoospermia | 1G2 | | Negative | 9.2 | 7.9 | NA |
| 11 | 28 | 7 | 11 | 28 | 61 | 3 | Oligoasthenoteratozoospermia | 2G1 | | Negative | 12.0 | 11.8 | NA |
| 12 | 29 | 12 | 15 | 27 | 58 | 11 | Oligoasthenospermia | 2G2 | | Negative | 9.3 | 13.2 | NA |
| 13 | 28 | 9 | 9 | 34 | 57 | 5 | Oligoasthenospermia | 2G2 | | Negative | 10.4 | 11.7 | NA |
| 14 | 28 | 6 | 9 | 11 | 80 | 4 | Oligoasthenospermia | 2G1 | | Negative | 11.6 | 12.8 | NA |
| 15 | 25 | 14 | 14 | 16 | 70 | 10 | Oligoasthenospermia | 1G2 | | Negative | 15.8 | 12.4 | NA |
| 16 | 26 | 9 | 9 | 42 | 49 | 4 | Oligoasthenospermia | 2G2 | | Negative | 8.9 | 8.8 | NA |
| 17 | 24 | 3 | 5 | 25 | 70 | 5 | Oligoasthenospermia | 2G2 | | Negative | 10.2 | 13.1 | NA |
| 18 | 22 | 14 | 18 | 36 | 46 | 4 | Oligoasthenospermia | 2G2 | | Negative | 12.4 | 14.7 | NA |
| 19 | 27 | 9 | 7 | 21 | 72 | 2 | Oligoasthenoteratozoospermia | 2G1 | | Negative | 13.3 | 10.2 | NA |
| 20 | 23 | 10 | 16 | 46 | 38 | 5 | Oligoasthenospermia | 2G2 | | Negative | 11.9 | 11.3 | NA |
| 21 | 27 | 15 | 21 | 21 | 58 | 6 | Asthenozoospermia | 2G2 | | Negative | 4.6 | 10.6 | NA |
| 22 | 24 | 5 | 12 | 38 | 50 | 2 | Oligoasthenoteratozoospermia | 2G3 | | Negative | 10.5 | 12.3 | NA |
| 23 | 28 | 30 | 28 | 35 | 37 | 4 | Asthenozoospermia | 2G1 | | Negative | 8.7 | 8.5 | NA |
| 24 | 24 | 14 | 18 | 34 | 48 | 3 | Oligoasthenoteratozoospermia | 2G1 | | Negative | 7.8 | 11.4 | NA |
| 25 | 21 | 5 | 8 | 23 | 69 | 2 | Oligoasthenoteratozoospermia | 2G2 | | Negative | 8.3 | 7.7 | NA |
| 26 | 25 | 18 | 21 | 20 | 59 | 3 | Asthenoteratozoospermia | 1G2 | | Negative | 8.6 | 12.9 | NA |
| 27 | 30 | 8 | 14 | 44 | 42 | 4 | Oligoasthenospermia | 2G2 | | Negative | 9.4 | 8.8 | NA |
| 28 | 25 | 5 | 9 | 40 | 51 | 2 | Oligoasthenoteratozoospermia | 2G4 | | Negative | 7.8 | 7.6 | NA |
| 29 | 28 | 13 | 10 | 30 | 60 | 1 | Oligoasthenoteratozoospermia | 2G4 | | Negative | 9.1 | 8.7 | NA |
| 30 | 23 | 6 | 3 | 51 | 46 | 4 | Oligoasthenospermia | 2G2 | | Negative | 11.7 | 11.5 | NA |
| 31 | 22 | 5 | 12 | 33 | 55 | 4 | Oligoasthenospermia | 2G2 | | Negative | 8.5 | 12.7 | NA |
| 32 | 21 | 8 | 4 | 21 | 75 | 2 | Oligoasthenoteratozoospermia | 2G2 | | Negative | 10.6 | 9.1 | NA |
| 33 | 22 | 6 | 2 | 44 | 54 | 5 | Oligoasthenospermia | 1G1/1G2 | | Negative | 12.7 | 8.3 | NA |
| 34 | 24 | 3 | 5 | 20 | 75 | 1 | Oligoasthenoteratozoospermia | 2G2 | | Negative | 8.6 | 13.2 | NA |
| 35 | 24 | 16 | 15 | 30 | 55 | 5 | Asthenozoospermia | 2G2 | | Negative | 4.3 | 14.8 | NA |
| 36 | 25 | 2 | 5 | 15 | 80 | 2 | Oligoasthenoteratozoospermia | 1G2 | | Negative | 10.1 | 10.6 | NA |
| 37 | 29 | 2 | 10 | 12 | 78 | 2 | Oligoasthenoteratozoospermia | 2G2 | | Negative | 9.4 | 12.2 | NA |
| 38 | 25 | 2 | 13 | 20 | 67 | 1 | Oligoasthenoteratozoospermia | 2G2 | | Negative | 12.1 | 18.7 | NA |
| 39 | 28 | 7 | 9 | 34 | 57 | 1 | Oligoasthenoteratozoospermia | 2G3 | | Negative | 6.3 | 6.3 | NA |
| 40 | 30 | 30 | 28 | 34 | 38 | 8 | Asthenozoospermia | 2G2 | | Negative | 8.4 | 12.8 | NA |
| 41 | 24 | 14 | 17 | 43 | 40 | 3 | Oligoasthenoteratozoospermia | 2G2 | | Negative | 12.6 | 10.5 | NA |
| 42 | 28 | 31 | 28 | 32 | 40 | 7 | Asthenozoospermia | 2G1 | | Positive | 5.2 | 7.4 | 1 Boy |
| 43 | 26 | 35 | 25 | 30 | 45 | 13 | Asthenozoospermia | 2G1 | | Positive | 5.4 | 9.3 | 1 Boy |
| 44 | 29 | 35 | 28 | 41 | 31 | 8 | Asthenozoospermia | 2G1 | | Positive | 5.7 | 3.4 | 1 Boy |
| 45 | 21 | 24 | 21 | 33 | 46 | 7 | Asthenozoospermia | 2G1 | | Positive | 3.9 | 4.4 | 1 Boy |
| 46 | 23 | 14 | 18 | 21 | 61 | 12 | Oligoasthenospermia | 2G1 | | Positive | 4.4 | 6.2 | 1 Boy |
| 47 | 25 | 28 | 21 | 32 | 47 | 12 | Asthenozoospermia | 2G1 | | Positive | 2.3 | 5.9 | 1 Boy |
| 48 | 26 | 15 | 18 | 50 | 32 | 8 | Asthenozoospermia | 2G1 | | Positive | 3.9 | 7.8 | 1 Boy |
| 49 | 23 | 21 | 21 | 38 | 41 | 6 | Asthenozoospermia | 2G1 | | Positive | 3.1 | 3.9 | 1 Boy |
| 50 | 24 | 4 | 12 | 18 | 70 | 7 | Oligoasthenospermia | 2G1 | | Positive | 4.2 | 4.1 | 1 Boy |
| 51 | 30 | 9 | 5 | 25 | 70 | 9 | Oligoasthenospermia | 2G1 | | Positive | 5.8 | 6.4 | 1 Boy |
| 52 | 29 | 31 | 19 | 35 | 46 | 12 | Asthenozoospermia | 2G1 | | Positive | 5.2 | 7.1 | Boy/Girl |
| 53 | 28 | 8 | 11 | 22 | 67 | 4 | Oligoasthenospermia | 2G1 | | Positive | 9.1 | 6.8 | 1 Girl |
| 54 | 26 | 15 | 22 | 32 | 46 | 5 | Asthenozoospermia | 2G1 | | Positive | 6.9 | 5.9 | 1 Girl |
| 55 | 23 | 17 | 21 | 25 | 54 | 7 | Asthenozoospermia | 2G1 | | Positive | 3.8 | 5.9 | 1 Girl |
| 56 | 30 | 9 | 12 | 19 | 69 | 4 | Oligoasthenospermia | 2G1 | | Positive | 12.3 | 6.8 | 1 Girl |
| 57 | 21 | 12 | 14 | 30 | 56 | 5 | Oligoasthenospermia | 2G1 | | Positive | 6.8 | 10.7 | 1 Girl |
| 58 | 24 | 25 | 28 | 34 | 38 | 7 | Asthenozoospermia | 2G1 | | Positive | 5.9 | 4.1 | 1 Girl |
| 59 | 25 | 15 | 26 | 32 | 42 | 6 | Asthenozoospermia | 3G1 | | Positive | 2.3 | 3.8 | 1 Girl |
| 60 | 27 | 18 | 13 | 37 | 50 | 3 | Asthenoteratozoospermia | 2G1 | | Positive | 5.1 | 2.2 | 1 Girl |
| 61 | 21 | 25 | 19 | 36 | 45 | 4 | Asthenozoospermia | 2G1 | | Positive | 3.3 | 5.1 | 1 Girl |
| 62 | 21 | 12 | 15 | 25 | 60 | 6 | Oligoasthenospermia | 2G1 | | Positive | 4.2 | 6.3 | 1 Girl |
| 63 | 24 | 25 | 21 | 26 | 53 | 12 | Asthenozoospermia | 2G1 | | Positive | 4.1 | 5.7 | 1 Girl |
| 64 | 26 | 13 | 13 | 15 | 72 | 5 | Oligoasthenospermia | 2G1 | | Positive | 3.9 | 7.1 | 1 Girl |
| 65 | 27 | 18 | 14 | 32 | 54 | 4 | Asthenozoospermia | 3G1 | | Positive | 6.8 | 4.8 | 2 Boy |
| 66 | 27 | 3 | 6 | 35 | 59 | 4 | Oligoasthenospermia | 2G1 | | Positive | 8.5 | 11.5 | 2 Boy |
| 67 | 24 | 4 | 8 | 14 | 78 | 4 | Oligoasthenospermia | 3G1 | | Positive | 7.4 | 7.7 | 2 Boy/Girl |
| 68 | 29 | 32 | 21 | 43 | 36 | 5 | Asthenozoospermia | 2G1 | | Positive | 6.6 | 8.9 | 2 Girl |
| 69 | 23 | 28 | 27 | 28 | 45 | 5 | Asthenozoospermia | 2G1 | | Positive | 6.8 | 4.9 | 2 Girl |
| 70 | 30 | 13 | 27 | 35 | 38 | 12 | Oligoasthenospermia | 2G1 | | Positive | 6.5 | 5.6 | 2 Girl |
| 71 | 21 | 15 | 19 | 35 | 46 | 5 | Asthenozoospermia | 2G1 | | Positive | 5.6 | 6.8 | 2 Girl |
| 72 | 23 | 21 | 22 | 34 | 44 | 3 | Asthenoteratozoospermia | 1G1/1G2 | | Positive | 6.6 | 10.3 | NA |
| 73 | 24 | 16 | 18 | 36 | 46 | 4 | Asthenozoospermia | 1G1/1G2 | | Positive | 5.2 | 7.8 | NA |
| 74 | 28 | 18 | 24 | 41 | 35 | 15 | Asthenozoospermia | 2G1 | | Positive | 7.7 | 8.2 | NA |
| 75 | 27 | 17 | 21 | 41 | 38 | 4 | Asthenozoospermia | 2G1 | | Positive | 8.2 | 5.1 | NA |
| 76 | 29 | 12 | 13 | 11 | 76 | 8 | Oligoasthenospermia | 2G1 | | Positive | 4.3 | 9.4 | NA |
| 77 | 22 | 24 | 19 | 33 | 48 | 6 | Asthenozoospermia | 2G1 | | Positive | 10.1 | 6.2 | NA |
| 78 | 29 | 17 | 19 | 32 | 49 | 5 | Asthenozoospermia | 2G1 | | Positive | 3.8 | 8.8 | NA |
| 79 | 21 | 24 | 23 | 36 | 41 | 8 | Asthenozoospermia | 2G1 | | Positive | 4.2 | 4.4 | NA |
| 80 | 22 | 16 | 16 | 43 | 41 | 8 | Asthenozoospermia | 2G1 | | Positive | 8.6 | 2.0 | NA |
| 81 | 24 | 18 | 14 | 38 | 48 | 3 | Asthenoteratozoospermia | 2G1 | | Positive | 6.2 | 4.5 | NA |
| 82 | 24 | 8 | 11 | 31 | 58 | 4 | Oligoasthenospermia | 1G1/1G2 | | Positive | 2.8 | 8.6 | NA |
| 83 | 29 | 12 | 6 | 9 | 85 | 5 | Oligoasthenospermia | 2G2 | | Positive | 11.7 | 10.7 | NA |
| 84 | 21 | 24 | 27 | 15 | 58 | 5 | Asthenozoospermia | 1G1/1G2 | | Positive | 3.6 | 7.6 | NA |
| 85 | 29 | 15 | 16 | 46 | 38 | 7 | Asthenozoospermia | 2G1 | | Positive | 6.8 | 8.5 | NA |
| Mean (± SD) | **25.52 (± 2.81)** | **17.74 (± 10.13)** | **13.80 (± 8.16)** | **28.95 (± 11.19)** | **58.55 (± 15.92)** | **7.36 (± 4.13)** | - | - | | - | **8.90 (± 4.05)** | **10.84 (± 4.76)** | - |

**Abbreviations:** Beta Human Chorionic Gonadotropin (β-hCG), Follicle-Stimulating Hormone (FSH), Luteinizing Hormone (LH), Grade 1 (G1), Grade 2 (G2), Not Available (NA)

**Supplementary Table 3|** Small RNA Quantification.

| Small RNAs | Group A | Group B | Group C |
| --- | --- | --- | --- |
| lncRNA | 651380 (71.57%) | 706321 (72.47%) | 882336 (74.08%) |
| rRNA | 89641 (9.85%) | 90201 (9.25%) | 86066 (7.23%) |
| piRNA | 33900 (3.72%) | 38748 (3.98%) | 71005 (5.96%) |
| sRNA | 23783 (2.61%) | 26571 (2.73%) | 34742 (2.92%) |
| tRNA | 40244 (4.42%) | 40610 (4.17%) | 39693 (3.33%) |
| ncRNA | 24673 (2.71%) | 24725 (2.54%) | 23780 (2.00%) |
| pre_miRNA | 13523 (1.49%) | 13596 (1.39%) | 14673 (1.23%) |
| miRNA | 7553 (0.83%) | 7403 (0.76%) | 7871 (0.66%) |
| misc_RNA | 5181 (0.57%) | 5589 (0.57%) | 6555 (0.55%) |
| SRP_RNA | 3630 (0.40%) | 4543 (0.47%) | 5900 (0.50%) |
| snRNA | 5578 (0.61%) | 5602 (0.57%) | 6123 (0.51%) |
| Y_RNA | 7389 (0.81%) | 6765 (0.69%) | 6389 (0.54%) |
| snoRNA | 2106 (0.23%) | 2315 (0.24%) | 3821 (0.32%) |
| circRNA | 643 (0.07%) | 683 (0.07%) | 980 (0.08%) |
| antisense_RNA | 252 (0.03%) | 288 (0.03%) | 353 (0.03%) |
| scaRNA | 139 (0.02%) | 137 (0.01%) | 196 (0.02%) |
| precursor_RNA | 149 (0.02%) | 156 (0.02%) | 184 (0.02%) |
| vault_RNA | 124 (0.01%) | 117 (0.01%) | 119 (0.01%) |
| RNase_MRP_RNA | 63 (0.01%) | 84 (0.01%) | 97 (0.01%) |
| other | 80 (0.01%) | 87 (0.01%) | 101 (0.01%) |
| scRNA | 63 (0.01%) | 72 (0.01%) | 49 (0.00%) |
| RNase_P_RNA | 42 (0.00%) | 44 (0.00%) | 48 (0.00%) |
| telomerase_RNA | 3 (0.00%) | 10 (0.00%) | 5 (0.00%) |
| tmRNA | 9 (0.00%) | 10 (0.00%) | 9 (0.00%) |
| hammerhead_ribozyme | 0 (0.00%) | 1 (0.00%) | 0 (0.00%) |
| autocatalytically_spliced_intron | 0 (0.00%) | 0 (0.00%) | 0 (0.00%) |
| guide_RNA | 3 (0.00%) | 1 (0.00%) | 2 (0.00%) |
| ribozyme | 2 (0.00%) | 0 (0.00%) | 0 (0.00%) |

- **Abbreviations:** lncRNA: Long non-coding RNA, rRNA: Ribosomal RNA, piRNA: Piwi-interacting RNA, sRNA: Small RNA, tRNA: Transfer RNA, ncRNA: Non-coding RNA, pre_miRNA: Precursor microRNA, miRNA: MicroRNA, misc_RNA: Miscellaneous RNA, SRP_RNA: Signal Recognition Particle RNA, snRNA: Small nuclear RNA, Y_RNA: Y RNA, snoRNA: Small nucleolar RNA, circRNA: Circular RNA, antisense_RNA: Antisense RNA, scaRNA: Small cajal body-specific RNA, precursor_RNA: Precursor RNA, vault_RNA: Vault RNA, RNase_MRP_RNA: RNase mitochondrial RNA processing RNA, scRNA: Small cytoplasmic RNA, RNase_P_RNA: RNase P RNA, telomerase_RNA: Telomerase RNA, tmRNA: Transfer-messenger RNA.
- **Group A:** Sperm with good motility and morphology (n=13).
- **Group B:** Sperm with very low or no motility but good morphology (n=13).
- **Group C:** Sperm with very low or no motility and abnormal morphology (n=13).
